# The calcium-sensing receptor modulates the prostaglandin E_2_ pathway in intestinal inflammation

**DOI:** 10.3389/fphar.2023.1151144

**Published:** 2023-04-20

**Authors:** Valeriya Gushchina, Nadja Kupper, Michael Schwarzkopf, Gitta Frisch, Karina Piatek, Cornelia Aigner, Alexandra Michel, Hemma Schueffl, Luca Iamartino, Taha Elajnaf, Teresa Manhardt, Andrea Vlasaty, Petra Heffeter, Marcella Bassetto, Enikö Kállay, Martin Schepelmann

**Affiliations:** ^1^ Institute for Pathophysiology and Allergy Research, Center of Pathophysiology, Infectiology and Immunology, Medical University of Vienna, Vienna, Austria; ^2^ Center for Cancer Research and Comprehensive Cancer Center, Medical University of Vienna, Vienna, Austria; ^3^ SiSaf Ltd, Guildford, United Kingdom; ^4^ Nuffield Department of Women’s and Reproductive Health, Medical Sciences Division, University of Oxford, Oxford, United Kingdom; ^5^ School of Pharmacy and Pharmaceutical Sciences, College of Biomedical and Life Sciences, Cardiff University, Cardiff, United Kingdom; ^6^ Department of Chemistry, Faculty of Science and Engineering, Swansea University, Swansea, United Kingdom

**Keywords:** calcium-sensing receptor (CaSR), prostaglandins, inflammation, colitis, calcimimetic, calcilytic

## Abstract

**Introduction:** The prostaglandin E_2_ (PGE_2_) pathway is one of the main mediators of intestinal inflammation. As activation of the calcium-sensing receptor (CaSR) induces expression of inflammatory markers in the colon, we assessed the impact of the CaSR on the PGE_2_ pathway regulation in colon cancer cells and the colon *in vitro* and *in vivo*.

**Methods and Results:** We treated CaSR-transfected HT29 and Caco-2 colon cancer cell lines with different orthosteric ligands or modulators of the CaSR and measured gene expression and PGE_2_ levels. In CaSR-transfected HT29^CaSR-GFP^ and Caco-2^CaSR-GFP^ cells, the orthosteric CaSR ligand spermine and the positive allosteric CaSR modulator NPS *R*-568 both induced an inflammatory state as measured by IL-8 gene expression and significantly increased the expression of the PGE_2_ pathway key enzymes cyclooxygenase (COX)-2 and/or prostaglandin E_2_ synthase 1 (PGES-1). Inhibition of the CaSR with the calcilytic NPS 2143 abolished the spermine- and NPS *R*-568-induced pro-inflammatory response. Interestingly, we observed cell-line specific responses as *e.g*. PGES-1 expression was affected only in HT29^CaSR-GFP^ but not in Caco-2^CaSR-GFP^ cells. Other genes involved in the PGE_2_ pathway (COX-1, or the PGE_2_ receptors) were not responsive to the treatment. None of the studied genes were affected by any CaSR agonist in GFP-only transfected HT29^GFP^ and Caco-2^GFP^ cells, indicating that the observed gene-inducing effects of spermine and *R*-568 were indeed mediated by the CaSR.

*In vivo*, we had previously determined that treatment with the clinically approved calcimimetic cinacalcet worsened symptoms in a dextran sulfate sodium (DSS)-induced colitis mouse model. In the colons of these mice, cinacalcet significantly induced gene expression of PGES-2 and the EP3 receptor, but not COX-2; while NPS 2143 increased the expression of the PGE_2_-degrading enzyme 15-hydroxyprostaglandin dehydrogenase (15-PGDH). Importantly, neither treatment had any effect on the colons of non-DSS treated mice.

**Discussion:** Overall, we show that activation of the CaSR induces the PGE_2_ pathway, albeit with differing effects *in vitro* and *in vivo*. This may be due to the different microenvironment *in vivo* compared to *in vitro*, specifically the presence of a CaSR-responsive immune system. Since calcilytics inhibit ligand-mediated CaSR signaling, they may be considered for novel therapies against inflammatory bowel disease.

## 1 Introduction

The calcium-sensing receptor (CaSR) is a versatile G protein-coupled receptor that is best known for its regulation of Ca^2+^ homeostasis in the parathyroid glands (Brown et al., 1993; Hannan et al., 2018).

Apart from Ca^2+^, the CaSR acts as a multimodal sensor for a plethora of other orthosteric agonists including divalent and trivalent cations, aromatic amino acids (*e.g.*, Phe, Trp), polyamines (*e.g.*, spermine), polypeptides, and even antibiotics (*e.g.*, neomycin), (Conigrave and Brown, 2006; Díaz-Soto et al., 2016). Because of this diversity in ligands, and its tendency to couple to numerous G proteins (G_q/11_, G_i/o_ and G_12/13_), the CaSR is involved in the initiation of multiple downstream signaling pathways by a process known as biased signaling (Leach et al., 2014). In addition to these natural ligands, small molecules, known as “calcimimetics” and “calcilytics,” have been developed that target the CaSR on its allosteric binding sites. NPS *R*-568 and cinacalcet are both calcimimetics, which bind to the receptor’s transmembrane domain and increase the sensitivity of the CaSR to its orthosteric ligands. Conversely, calcilytics, like NPS 2143, decrease the sensitivity of the CaSR to its ligands (Nemeth and Goodman, 2016).

The CaSR is expressed also in tissues outside of the Ca^2+^ homeostatic system, such as the vasculature, the lung and the intestine. In these tissues, the CaSR regulates a range of physiological processes which are responsible for vascular tone and mineral homeostasis (Schepelmann et al., 2016; Schepelmann et al., 2022), intestinal fluid secretion and water transport, epithelial layer integrity, inflammation, as well as proliferation, differentiation, and apoptosis, as reviewed in (Hannan et al., 2018; Iamartino et al., 2018).

The role of the CaSR in the intestines is not quite understood yet. While previous studies suggested an anti-inflammatory role for the CaSR in the colon (Cheng et al., 2014; Zhang et al., 2015a; Mine and Zhang, 2015), we have recently found that CaSR activation with calcimimetics (NPS *R*-568, and cinacalcet) promoted while calcilytics (NPS 2143) inhibited intestinal inflammation (Elajnaf et al., 2019; Iamartino et al., 2020; Schepelmann et al., 2021). We thus hypothesized that calcilytics may constitute a novel therapy for inflammatory bowel disease (IBD), just as in the lungs, where calcilytics are proposed as a safe and effective asthma treatment (Yarova et al., 2015; Yarova et al., 2021).

One of the central pathways involved in intestinal inflammation is the prostaglandin E_2_ (PGE_2_) pathway. Cyclooxygenases (COX) catalyze the rate-limiting steps of PGE_2_ synthesis, which is the conversion of arachidonic acid to PGH_2_. There are two primary isomers of COX. COX-1 is constitutively expressed and plays a role in tissue homeostasis. COX-2 is inducible and upregulated by various extracellular and intracellular stimuli, *e.g.*, lipopolysaccharides, interleukin-1, tumor necrosis factor, epidermal growth factor, or oncogenes. PGH_2_ is then isomerized to PGE_2_ by one of the isoforms of prostaglandin E synthases (PGES). Secreted PGE_2_ can then bind to one of the four extracellular PGE_2_ receptors (EP1-4), (Greenhough et al., 2009). The accumulation of COX-2-derived PGE_2_ is subject to the upregulation of several signaling pathways and downregulation of apoptotic proteins (Gandhi et al., 2017). Thus, COX-2 over-expression contributes to inflammation, hence colitis and colitis-associated colorectal cancer. 15-Hydroxyprostaglandin dehydrogenase (15-PGDH) in turn inhibits prostaglandin signaling by degrading PGE_2_.

We have previously found that CaSR activation with a calcimimetic worsened clinical symptoms in a mouse model of colitis (Elajnaf et al., 2019), and upregulated various inflammatory pathways in CaSR-transfected human colorectal cancer cells (Iamartino et al., 2020). In the present study, we therefore aimed to determine the effect of the CaSR on the PGE_2_ pathway both *in vitro* and *in vivo*. We were able to show that activation of the CaSR induced gene expression of several PGE_2_ pathway genes as well as PGE_2_ secretion in a ligand-dependent manner, and that this effect could be prevented by calcilytics.

## 2 Materials and methods

All solvents and reagents were obtained from Merck (United States), unless otherwise stated. CaSR allosteric modulators cinacalcet, NPS *R*-568 and NPS 2143 were obtained from Tocris Bioscience (United Kingdom). GSK3004774 was custom synthetized previously (Elajnaf et al., 2019).

### 2.1 Colon cancer cells

We used two colon cancer cell lines which were lentivirally transduced to stably express the CaSR fused to GFP (HT29^CaSR−GFP^; Caco-2^CaSR−GFP^) or GFP alone as control (HT29^GFP^; Caco-2^GFP^), (Iamartino et al., 2020).

Both cell lines were cultured in Dulbecco’s Modified Eagle’s Medium (DMEM) medium, supplemented with 10% fetal calf serum (FCS), 10 mM HEPES buffer solution, 2 mM L-Glutamine, and 100 U/mL Pen-Strep (all Thermo Fischer Scientific, Gibco, United States). Cells were maintained at 37°C in a humid atmosphere with 5% CO_2_. Passaging occurred once a week, while selection for the stably transfected cells with puromycin occurred every 4 weeks. Once the cells reached 80% confluency, they were treated with vehicle (DMSO or H_2_O), different orthosteric ligands (5 mM spermine, 300 µM neomycin, 1 mM L-Phe, or 1 mM L-Trp) or allosteric modulators of the CaSR (1 µM NPS *R*-568, 10 µM GSK3004774, or NPS 2143), (for exact treatments and their concentrations, see individual figures). Concentrations of the orthosteric ligands were chosen based on the respective publications (Quinn et al., 1997; Ziegelstein et al., 2006; Mine and Zhang, 2015). The final concentration of DMSO was 0.1% (*v*/*v*) in all conditions.

### 2.2 Animal tissues

For our *in vivo* experiments, we analyzed gene expression from colons of 8-week old female Balb/C mice with chemically induced colitis, treated with allosteric CaSR modulators (cinacalcet, GSK3004774, or NPS 2143; 10 mg/kg) or vehicle (20% cyclodextrin) by oral gavage for 2 weeks from a previous study (Elajnaf et al., 2019). In that experiment, colitis had been chemically induced by administration of 3.5% dextran sulphate sodium (DSS) in drinking water over 7 days (see Elajnaf et al., 2019 for experimental and treatment details). The non-colitis group received CaSR modulators (cinacalcet or NPS 2143; 30 mg/kg) or vehicle (20% cyclodextrin) for 2 weeks. Pieces of max. 20 mg of the proximal and distal colon were used for RNA isolation. All animal experiments were approved by the Ethics Committee of the Medical University of Vienna and the Austrian Federal Ministry of Education, Science and Research (BMWFW-66.009/0401-WF/V/3b/2017, BMWFW-66.009/0006-WF/V/3b/2017 and BMBWF-66.009/0394-V/3b/2018) and carried out in accordance with the European Union Regulations on Care and Use of Laboratory Animals.

### 2.3 RNA isolation and RT-qPCR

Total RNA was isolated using TRIzol reagent (Invitrogen, United States) or EXTRAzol reagent (Blirt, PL) for cells and ReliaPrep RNA Miniprep Systems (Promega, United States) for homogenized colon tissue, following the respective manufacturer’s protocol. 1 μg of total RNA was used for cDNA synthesis using the High-Capacity RNA-to-cDNA Kit (Thermo Fischer Scientific, Applied Biosystems, United States). Obtained cDNA was used for reverse transcription quantitative PCR (RT-qPCR). RT-qPCR reactions were performed with Power SYBR Green PCR Master Mix (Thermo Fischer Scientific, Applied Biosystems). The primers were purchased from Merck and are listed in Supplementary Table S1. Results were normalized to the average of either hRPLP0 & hB2m (*in vitro* experiments), or mEef1β2 and mβ-actin (*in vivo* experiments) as housekeeping genes, using either commercially available human total RNA (Takara, Kusatsu, Japan) or mouse colon RNA (Takara, ClonTech, Japan) as calibrator. Relative gene expression was then determined using the ΔΔCT method.

### 2.4 Prostaglandin E_2_ assay

Once cells reached 80% confluency, they were treated with either CaSR allosteric modulators (single treatment or double treatment) or vehicle control. After 4 h the supernatant was collected and PGE_2_ concentration was measured using the Prostaglandin E_2_ Parameter Assay Kit (R&D Systems, Catalog #KGE004B, United States), following the manufacturer’s protocol.

### 2.5 Statistical analysis

Statistical analysis and visualization were performed using GraphPad Prism 9.3.1 software (San Diego, CA, United States) and RStudio 2022.07.2 (Boston, MA, United States). Results are presented as mean ± standard deviation. Data were analyzed by one-way ANOVA or Mann-Whitney *U* test; *p* < 0.05 was considered statistically significant. The specific statistical tests for each dataset are indicated in each respective figure and table legends.

## 3 Results

### 3.1 Activation of the CaSR leads to PGE_2_-pathway induction in colon cancer cells

Because CaSR expression is lost in colon cancer cell lines and reliable “normal” colon epithelial cell lines are not available, we used CaSR-transfected (HT29^CaSR−GFP^ and Caco-2^CaSR−GFP^) or empty vector-transfected (HT29^GFP^ and Caco-2^GFP^) colorectal cancer cells as model systems for our *in vitro* experiments, as previously described (Iamartino et al., 2020). In previous studies, we have already shown that calcimimetics induced inflammatory gene expression via the CaSR (Iamartino et al., 2020; Schepelmann et al., 2021). The gastrointestinal tract is rich in nutrients of which some are known CaSR ligands. As cellular responses mediated by the CaSR are highly ligand-dependent, we assessed the effect of selected physiological orthosteric CaSR ligands on the PGE_2_ pathway. To confirm that the selected ligands indeed elicit inflammatory response in the cells, we first measured their effect on the expression of the inflammatory marker interleukin 8 (IL-8). The allosteric CaSR activators (calcimimetics) NPS *R*-568 and GSK3004774 (Sparks et al., 2017; Elajnaf et al., 2019) were used as positive controls for CaSR-specific inflammatory gene induction (Schepelmann et al., 2021).

**FIGURE 1:**
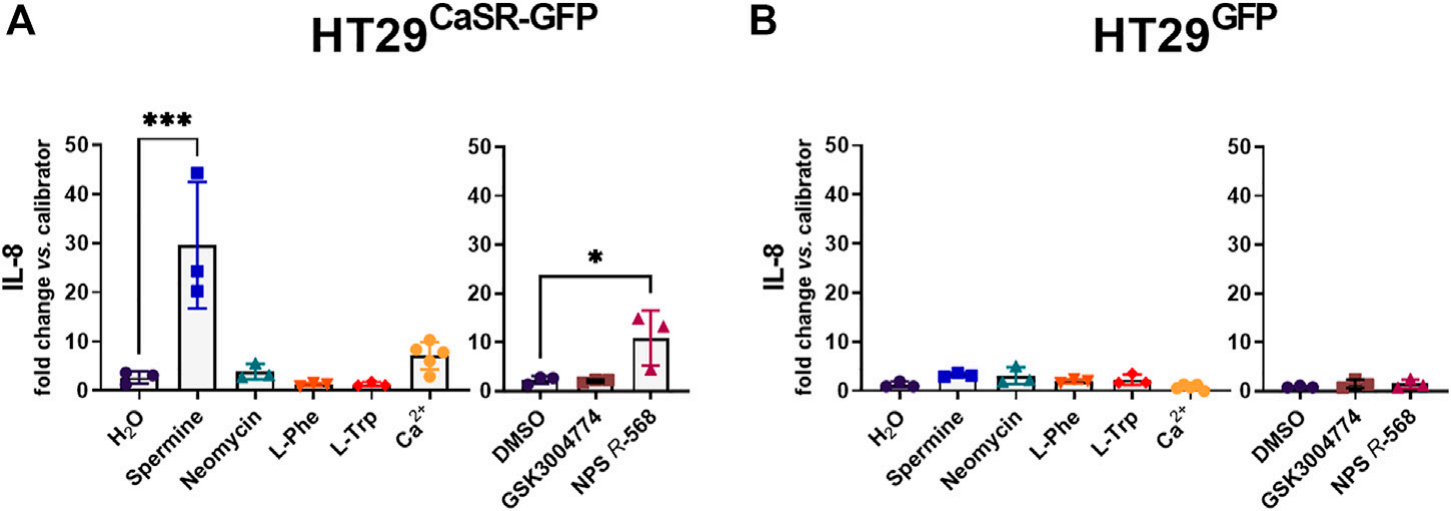
Induction of an inflammatory response in HT29 cells via the CaSR is ligand dependent. Relative gene expression of IL-8 in **(A)** HT29^CaSR−GFP^ and **(B)** HT29^GFP^ cells after 4 h treatment with CaSR ligands (left panel: H_2_O as vehicle control, 5 mM spermine, 300 µM neomycin, 1 mM L-Phe, 1 mM L-Trp, and 5 mM Ca^2+^) or CaSR modulators (right panel: 0.1% DMSO as vehicle control, 10 µM GSK3004774, and 1 µM NPS *R*-568). Mean ± SD, N = 3–5, one-way ANOVA with Dunnett’s *post hoc* test vs. vehicle control (H_2_O or 0.1% DMSO), *p < 0.05, ***p < 0.001.

Treatment with spermine and NPS *R*-568 significantly upregulated IL-8 expression (11.1-fold and 4.8-fold, respectively) in HT29^CaSR−GFP^ cells, while treatment with neomycin, L-Phe, L-Trp or GSK3004774 did not affect the expression of IL-8 (Figure 1A). Higher concentrations of L-Phe and L-Trp (5 mM and 10 mM) were also unable to upregulate IL-8 (Supplementary Table S2). None of the treatments had an effect on IL-8 gene expression in HT29^GFP^ cells (Figure 1B).

**FIGURE 2:**
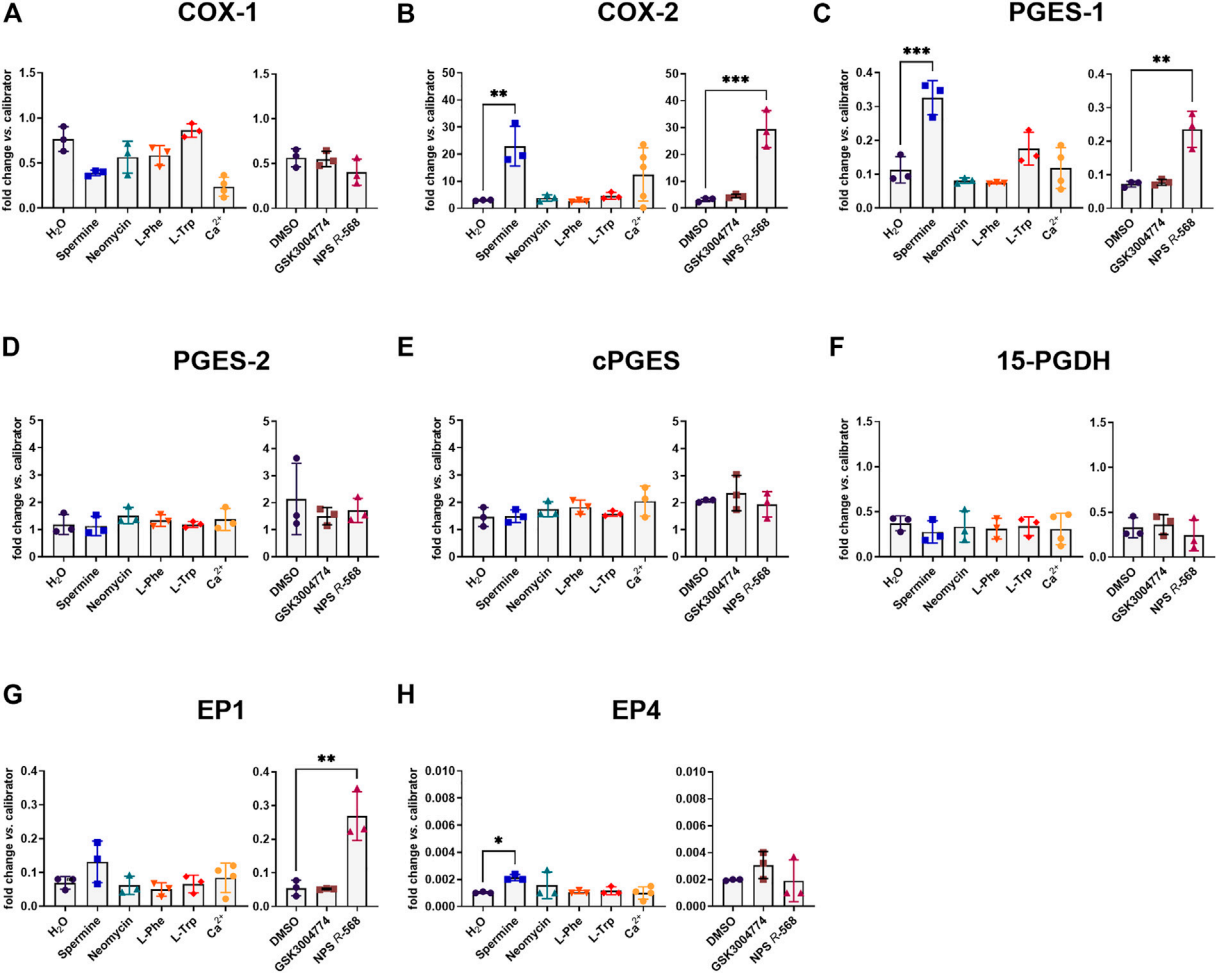
Induction of PGE_2_ pathway genes by CaSR ligands in HT29^CaSR−GFP^ cells. Relative gene expression of **(A)** COX-1, **(B)** COX-2, **(C)** PGES-1, **(D)** PGES-2, **(E)** cPGES, **(F)** 15-PGDH, **(G)** EP1, and **(H)** EP4 in HT29^CaSR−GFP^ cells after 4 h treatment with CaSR ligands (left panel: H_2_O as vehicle control, 5 mM spermine, 300 µM neomycin, 1 mM L-Phe, 1 mM L-Trp, and 5 mM Ca^2+^) or CaSR modulators (right panel: 0.1% DMSO as vehicle control, 10 µM GSK3004774, and 1 µM NPS *R*-568). Mean ± SD, N = 3–5, one-way ANOVA with Dunnett’s *post hoc* test vs. vehicle control (H_2_O or 0.1% DMSO), **p < 0.01, ***p < 0.001.

We then assessed the effect of the ligands on the main genes constituting the PGE_2_ pathway (see introduction). As mentioned above, the PGE_2_ pathway plays a pivotal role in intestinal inflammation. Stimulating the CaSR with the orthosteric agonist spermine or the positive allosteric modulator NPS *R*-568 resulted in the same pattern for COX-2 and PGES-1 expression as for IL-8 expression. In HT29^CaSR−GFP^ cells, both spermine and NPS *R*-568 significantly upregulated the expression of COX-2 (7.6-fold and 9.3-fold, respectively), (Figure 2B), and PGES-1 (2.9-fold and 3.3-fold, respectively), (Figure 2C). NPS *R*-568 upregulated the expression of EP1, while spermine upregulated EP4 expression (4.9-fold and 2.1-fold, respectively), (Figures 2G, H). Other genes involved in the PGE_2_ pathway were not affected (Figures 2A, D–F). In HT29^GFP^ cells, the treatments had no significant effect on the expression of the PGE_2_ pathway genes (Supplementary Figure S1), indicating the presence of the CaSR to be necessary for both spermine- and NPS *R*-568-induced gene expression.

We also assessed the mRNA levels of the CaSR gene, as it has been suggested that the expression is increased upon activation of the CaSR due to a process called agonist-driven insertional signaling (ADIS), (Grant et al., 2011). Treatment of HT29^CaSR−GFP^ cells with both spermine and NPS *R*-568 upregulated CaSR gene expression (4.6-fold and 5.2-fold, respectively). Elevated extracellular Ca^2+^ (5 mM) also led to a significant increase of CaSR gene expression (2.9-fold), (Supplementary Figure S2A). Again, no effect was observed in the HT29^GFP^ cells, where CaSR expression was close to undetectable under all conditions (Supplementary Figure S2B).

All numerical results are provided in Supplementary Table S2. The normalized expression of all investigated genes in the HT29^CaSR−GFP^ and HT29^GFP^ cells are visualized in Supplementary Figure S3, demonstrating the dependence of gene expression on the individual ligand and presence of the CaSR.

### 3.2 NPS 2143 inhibits spermine-induced inflammation

**FIGURE 3:**
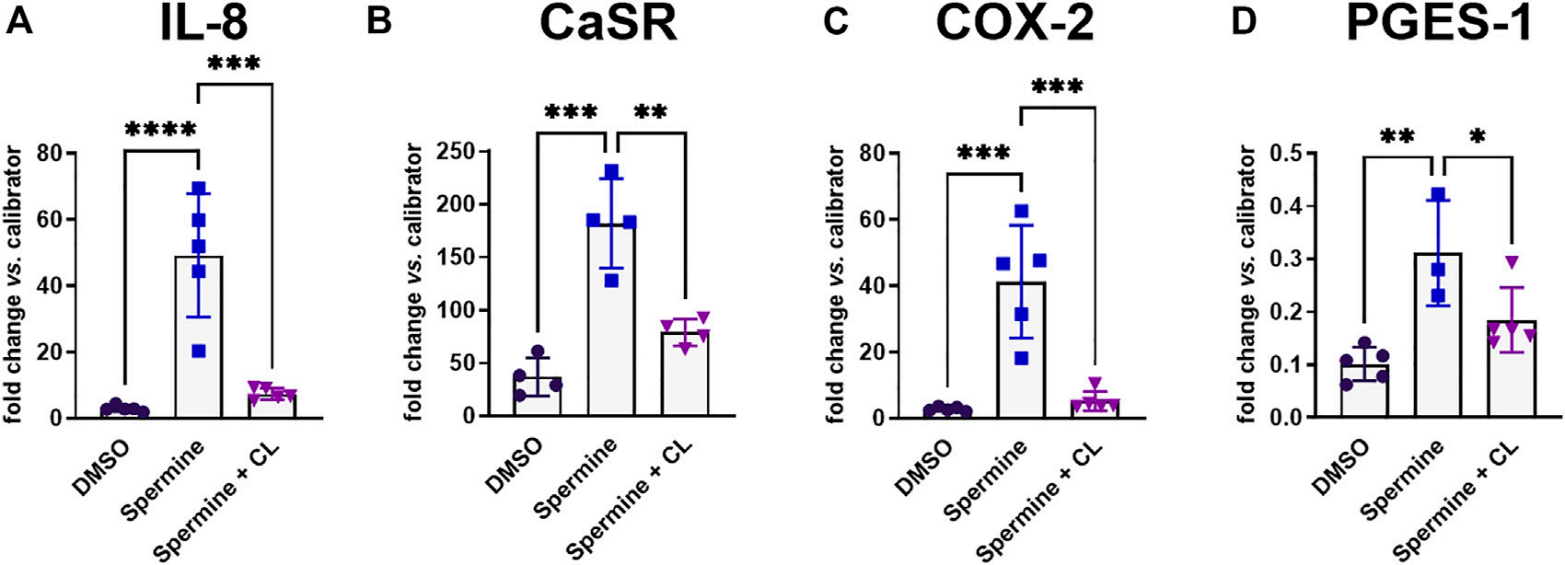
Gene induction by spermine can be suppressed by calcilytics. Relative gene expression of **(A)** IL-8, **(B)** CaSR, **(C)** COX-2, and **(D)** PGES-1 in HT29^CaSR−GFP^ cells after treatment with 5 mM spermine alone or together with the calcilytic 1 µM NPS 2143 (CL) for 4 h (0.1% DMSO as vehicle control). Mean ± SD, N = 3–5, one-way ANOVA with Tukey’s *post hoc* test (0.1% DMSO), *p < 0.05, **p < 0.01, ***p < 0.001, ****p < 0.0001.

Previous experiments have shown that pre-treatment with the calcilytic NPS 2143 prevented the induction of CaSR and IL-8 gene expression by the allosteric CaSR activator NPS *R*-568 (Schepelmann et al., 2021). We now found that pre-treatment of HT29^CaSR−GFP^ cells with NPS 2143 also inhibited the upregulation of IL-8, CaSR, COX-2 and PGES-1 induced by the orthosteric CaSR-agonist spermine (Figures 3A–D).

### 3.3 Secretion of PGE_2_ is dependent on CaSR-activation

**FIGURE 4:**
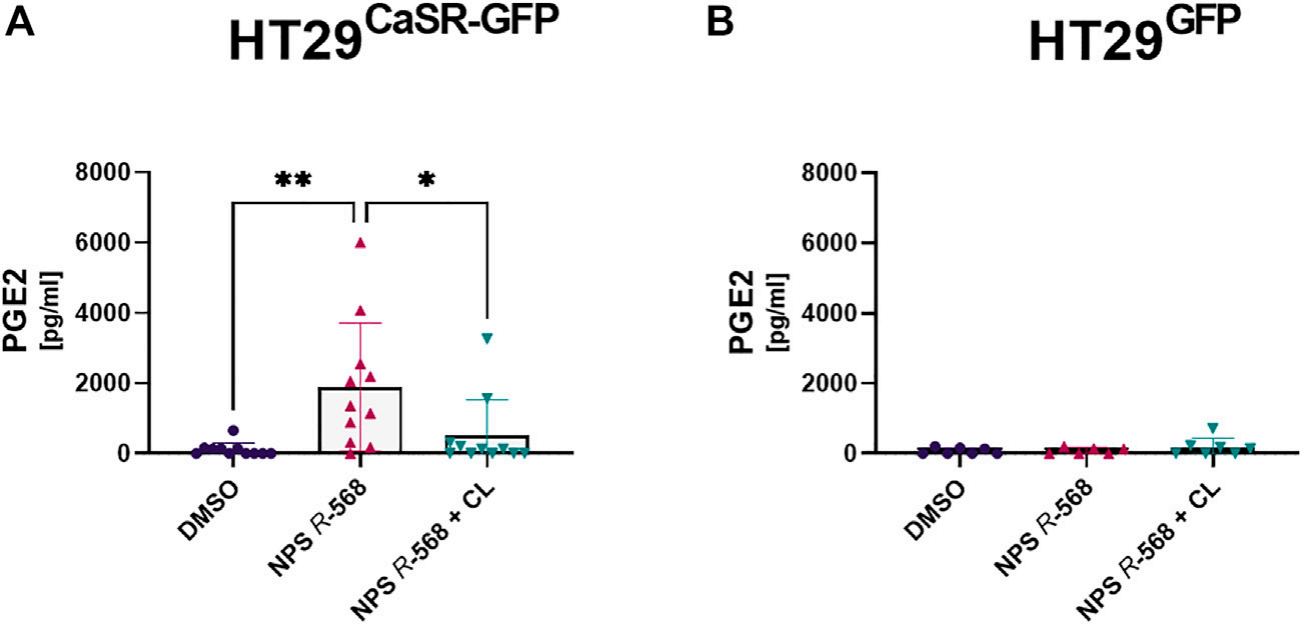
Induction and suppression of PGE_2_ secretion by CaSR activation and inhibition. PGE_2_ secretion in supernatant of **(A)** HT29^CaSR−GFP^ and **(B)** HT29^GFP^ after 4 h treatment with either vehicle control (0.1% DMSO), 1 µM NPS *R*-568 alone, or together with 1 µM of the calcilytic NPS 2143 (CL). Mean ± SD, N = 6–11, one-way ANOVA with Tukey’s *post hoc* test (0.1% DMSO), *p < 0.05, **p < 0.01.

As we had observed a significant upregulation of COX-2 and mPGES-1 gene expression, we also assessed whether this translated into changes in secretion of PGE_2_ by the cells. Treatment with NPS *R*-568 for 4 h increased PGE_2_ secretion (17.1-fold) in HT29^CaSR−GFP^ cells (Figure 4A), compared with vehicle, while pre-treatment with NPS 2143 inhibited the NPS *R*-568-induced PGE_2_ secretion. No effect of NPS *R*-568 treatment on PGE_2_ secretion was observed in HT29^GFP^ cells (Figure 4B).

### 3.4 Presence of the CaSR sensitizes cells to IL-1β-induced inflammation

**FIGURE 5:**
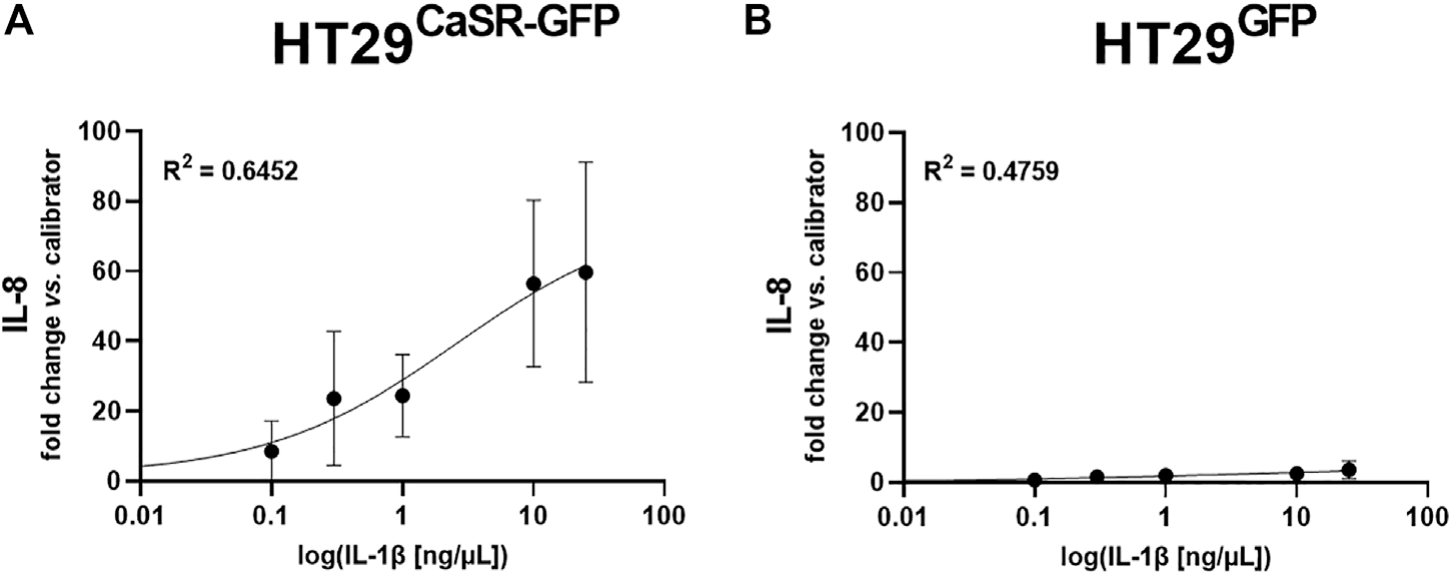
Induction of IL-8 gene expression via IL-1β is enhanced when the CaSR is present. Dose-response curve of relative gene expression of IL-8 in **(A)** HT29^CaSR−GFP^ cells and **(B)** HT29^GFP^ cells after 4 h treatment with IL-1β (0.1 ng/μL–25 ng/μL). Mean ± SD, N = 3–4. Sigmoidal 4-parameter fit, 0-concentration set to 10^−20^ ng/μL for logarithmic curve fitting. Calculated EC50 equals 2.49 ± 1.27 ng/μL.

As we had determined that both the orthosteric CaSR agonist spermine and the allosteric positive CaSR modulator NPS *R*-568 induced inflammatory responses in the HT29^CaSR−GFP^ but not the HT29^GFP^ cells, we were wondering whether the presence of the CaSR alone was also enough to increase susceptibility of the cells to inflammatory responses. Thus, we induced an inflammatory response in the cells using increasing concentrations (0.1 ng/μL–25 ng/μL) of the well-known inflammatory cytokine IL-1β, and measured IL-8 gene expression. IL-1β induced a strong dose-dependent increase of IL-8 only in HT29^CaSR−GFP^ cells (Figure 5A), while a much weaker effect was observed in HT29^GFP^ cells (Figure 5B).

### 3.5 Effect of CaSR activation in Caco-2 cells

**FIGURE 6:**
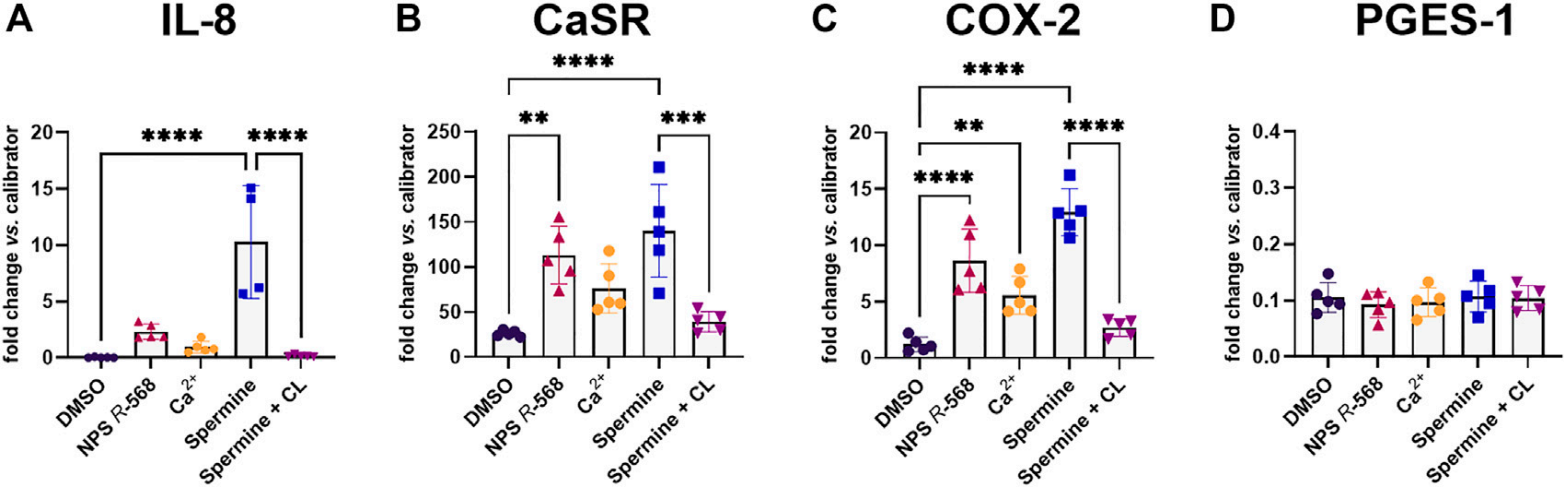
Gene induction and suppression by CaSR ligands in Caco-2^CaSR−GFP cells^. Relative gene expression of **(A)** IL-8, **(B)** CaSR, **(C)** COX-2, and **(D)** PGES-1 in Caco-2^CaSR−GFP^ cells after 4 h treatment with either vehicle control (0.1% DMSO), 1 µM NPS *R*-568, CaSR ligands (5 mM Ca^2+^, and 5 mM spermine), or 5 mM spermine in combination with 1 µM NPS 2143 (CL). Mean ± SD, N = 3–5, one-way ANOVA with Tukey’s *post hoc* test (0.1% DMSO), **p < 0.01, ***p < 0.001, ****p < 0.0001.

To confirm our results, we repeated the experiments in CaSR and empty-vector transfected Caco-2 cells. Caco-2^CaSR−GFP^ cells responded to the treatment with CaSR activators in a similar manner as HT29^CaSR−GFP^ cells. Both NPS *R*-568 and spermine upregulated CaSR, and COX-2 expression, while IL-8 was affected significantly only by spermine but not NPS *R*-568 (Figures 6A–C). Treatment of Caco-2^CaSR−GFP^ cells with Ca^2+^ upregulated only COX-2 gene expression, which in HT29^CaSR−GFP^ cells affected only the expression of the CaSR itself (the observed upregulation of the CaSR in Caco-2^CaSR−GFP^ following Ca^2+^ treatment Cells did not reach statistical significance with *P* ∼ 0.1). In Caco-2^CaSR−GFP^ cells, pre-treatment with NPS 2143 was also successful in abolishing spermine-induced (inflammatory) gene expression. We also noticed that the expression levels of all affected genes were slightly lower in Caco-2^CaSR−GFP^ cells than in the HT29^CaSR−GFP^ cells. In contrast to the HT29^CaSR−GFP^ cells, none of the treatments had any effect on PGES-1 gene expression in Caco-2^CaSR−GFP^ cells (Figure 6D). As in the HT29^GFP^ cells, the treatments had no effect on the gene expression of any of the selected targets in Caco-2^GFP^ cells (Supplementary Figure S4). PGE_2_ secretion was nearly undetectable both in Caco-2^CaSR−GFP^ and in Caco-2^GFP^ cells. Compared with Caco-2^GFP^ cells, treatment with NPS *R*-568 induced a slight elevation of PGE_2_ secretion by the Caco-2^CaSR−GFP^ cells, but this did not reach statistical significance (Supplementary Figure S5). All numerical results for Caco-2^CaSR−GFP^ and Caco-2^GFP^ cells are presented in Supplementary Table S3.

### 3.6 CaSR modulation influences PGE_2_ pathway in a mouse model of colitis

We next tested whether the PGE_2_ pathway would also be affected by modulation of the CaSR *in vivo.* Thus, we measured gene expression levels of CaSR and several PGE_2_ pathway genes in non-inflamed mouse colons, and in the inflamed colons of mice from our previous study (Elajnaf et al., 2019), which had been treated with different CaSR modulators. Due to differences in gene expression, molecular patterns, and morphology between the proximal and distal colon (Goldstone et al., 2012; Missiaglia et al., 2014), we analyzed both parts of the colon separately.

**FIGURE 7:**
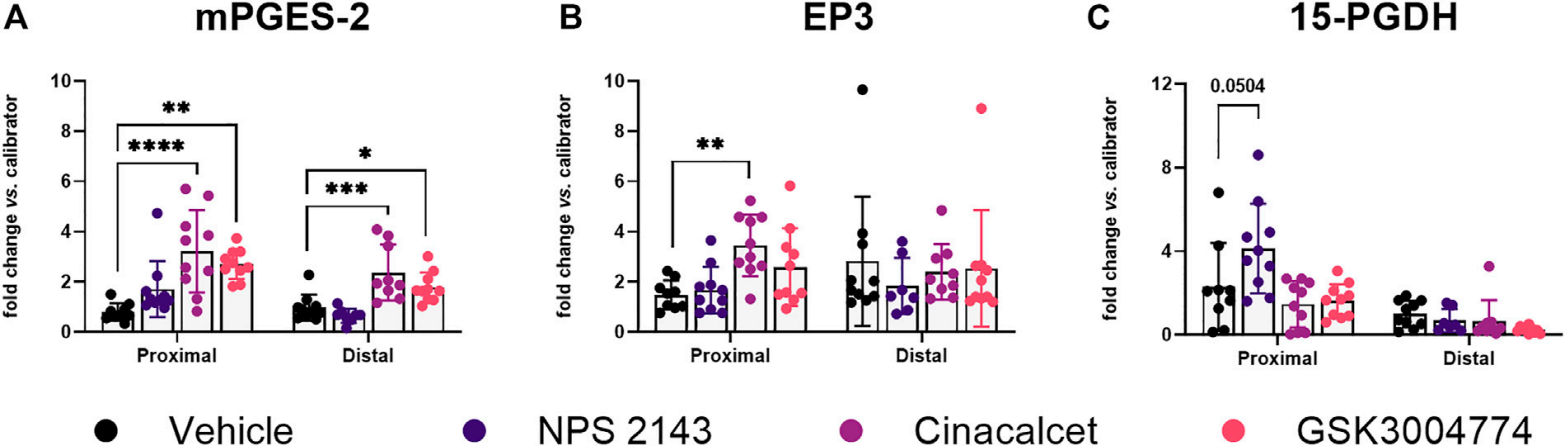
Altered PGE_2_ pathway genes in CaSR-ligand treated mice with colitis. Relative gene expression of **(A)** mPGES-2, **(B)** EP3, and **(C)** 15-PGDH in the colons of mice with DSS-induced colitis treated with CaSR allosteric modulators (each 10 mg/kg). Mean ± SD, N = 7–10. Proximal and distal colon were analyzed separately by one-way ANOVA with Dunnett’s *post hoc* test vs. vehicle control (20% cyclodextrin), *p < 0.05, **p < 0.01, ***p < 0.001, ****p < 0.0001.

CaSR gene expression was detected in all samples, but was unaffected by the treatment of the mice with CaSR modulators. In the inflamed colons the calcimimetics, cinacalcet and the non-gut-absorbable GSK3004774 (each 10 mg/kg), significantly increased expression of mPGES-2 in both the proximal and distal colon (Figure 7A). Cinacalcet also induced EP3 expression in the proximal colon (Figure 7B). We also observed a higher expression level of the PGE_2_ degrading enzyme 15-PGDH in the proximal colon after treatment with 10 mg/kg NPS 2143 (Figure 7C). Other genes of the PGE_2_ pathway were not affected (Supplementary Figure S6). In the non-inflamed colons, none of the CaSR modulators, even though administered at 3x higher concentration, affected gene expression of any of the investigated PGE_2_ pathway genes (Supplementary Figure S7). All numerical results are provided in Supplementary Table S4.

## 4 Discussion

Recently, we have proposed a pro-inflammatory role of the CaSR in the intestine, as stimulation of the CaSR in transfected colon cancer cells with positive CaSR allosteric modulators induced the expression of genes involved in several inflammatory pathways (Iamartino et al., 2020). In the current study, we have now shown that the PGE_2_ pathway is indeed one of the involved pathways.

Both the orthosteric ligand spermine, and the allosteric modulator NPS *R*-568 increased COX-2 and PGES-1 expression in HT29^CaSR−GFP^ cells, but not in HT29^GFP^ cells, once again marking the CaSR as the mediator of these pro-inflammatory effects. It has been shown previously that COX-2 and PGES-1 are upregulated in the inflamed intestinal tissue and in colorectal tumors (Williams et al., 1999; Yoshimatsu et al., 2001; Wang and DuBois, 2010). Both COX and the PGES isoforms are enzymes involved in the synthesis of PGE_2_. COX catalyzes the first step of PGE_2_ synthesis, that is, the conversion of arachidonic acid to PGH_2_. PGES subsequently isomerizes PGH_2_ to PGE_2_. The combined increase in expression of both genes eventually leads to increased PGE_2_ production and secretion. Secreted PGE_2_ then binds to one of the 4 PGE_2_ receptors (EP1–EP4) which determines cellular response (Greenhough et al., 2009). The CaSR affects the PGE_2_ pathway in a cell-line specific manner, as we have observed some distinct differences in the effect of the CaSR modulators between HT29^CaSR−GFP^ and Caco-2^CaSR−GFP^ cells. In contrast to HT29^CaSR−GFP^ cells, Caco-2^CaSR−GFP^ cells responded to CaSR stimulation only with COX-2 upregulation, while neither PGES-1 expression nor PGE_2_ production was altered significantly. COX-2 and PGES are often expressed at different levels in individual colorectal tumors, possibly due to different expression control mechanisms (Yoshimatsu et al., 2001). Although both HT29 and Caco-2 cells are derived from colorectal adenocarcinomas, they differ in genetic background and their potential to differentiate. It is thus possible that Caco-2 cells lack certain downstream effectors of the CaSR which are essential for PGES-1 upregulation, or activate different signaling pathways that do not lead to PGES-1 upregulation. The lack of PGES-1 induction may also explain why in Caco-2^CaSR−GFP^ cells PGE_2_ production was not increased. Even though both COX-2 and PGES-1 contribute to PGE_2_ production, it was suggested that activation of PGES-1 is more closely related to PGE_2_ induction in colon cancer (Kim and Kim, 2022). As shown before (Iamartino et al., 2020; Schepelmann et al., 2021), pre-incubation with the calcilytic NPS 2143 significantly reduced the NPS *R*-568-, and thus CaSR-induced gene expressions, as demonstrated before, indicating that the observed responses really were mediated via CaSR stimulation rather than unspecific actions of the tested ligands.

Spermine was identified as an orthosteric ligand of the CaSR already in 1997, shortly after the receptor’s first cloning (Quinn et al., 1997). Polyamines such as spermine, found in our daily diet, or produced by intestinal microbiota, are involved in the maintenance of proper gastrointestinal function (Pegg, 2014; Liu et al., 2022). The role of spermine seems to be multifold: spermine supports the barrier function of the gut, induces gut maturation and increases longevity (Bekebrede et al., 2020). It also suppresses cytokine synthesis in macrophages, preventing tissue damage-associated inflammation (Zhang et al., 2000). On the other hand, when acting as agonist of the CaSR, we have now demonstrated that spermine induces pro-inflammatory marker expression in the colon cells *in vitro* which can be inhibited by pre-incubation of the cells with the negative CaSR allosteric modulator NPS 2143. A similar situation has been observed in the lung. While some reports suggest an anti-inflammatory effect of spermine in the lungs (Wawrzyniak et al., 2021), spermine and other polyamines were shown to promote increased pulmonary fibrosis, airway hyperresponsiveness and asthma severity by activating the CaSR expressed in airway smooth muscle cells and epithelial cells. Calcilytics are able to prevent these CaSR-induced effects (Yarova et al., 2015; Wolffs et al., 2019). Here, we have demonstrated that calcilytics can exert the same effect also in the colon.

Interestingly, of the tested orthosteric CaSR ligands, only spermine affected gene expression, while *i.a.* the aromatic amino acids had no effect. Amino acids elicit slightly different intracellular signaling responses from the CaSR than spermine which may account for this difference. It is also possible that the colon cells simply would not be sensitive to amino acids as they would be constantly exposed to them in the form of the passing chyme. An important factor to take into consideration in these studies is the absence of an immune system within the highly artificial *in vitro* environment which consists only of a single type of cell.

The role of the CaSR in inflammation is very complex. Amongst the directly affected tissues, it is also present on a variety of immune cells (Iamartino and Brandi, 2022). We have demonstrated previously that the immune system in the colon is directly affected by CaSR modulators (Elajnaf et al., 2019). In that study we had used the DSS-induced colitis model of IBD, as it resembles human ulcerative colitis (Chassaing et al., 2014). While treatment with NPS 2143 reduced some of the colitis symptoms, the calcimimetic cinacalcet exhibited a rather pro-inflammatory effect. In the present study, we now tested whether activation of the PGE_2_ pathway is affected in the observed calcimimetic-mediated increase of disease severity, as suggested by the results from the transfected colon cancer cells. Interestingly, in the inflamed colons, treatment with the CaSR allosteric modulators did indeed affect the PGE_2_ pathway, but not via the expression of COX-2 (in contrast to the *in vitro* results where COX-2 was significantly upregulated in both HT29^CaSR−GFP^ and Caco-2^CaSR−GFP^ cells after stimulation of the CaSR).

Instead, stimulation of the CaSR *in vivo* affected the PGE_2_ pathway by upregulation of mPGES-2 and EP3. Treatment with both cinacalcet and GSK3004774 significantly increased the expression of mPGES-2 in the proximal and distal colon, while mPGES-1 was not affected at all. mPGES-1 is a highly inducible isoform of PGES, while the other two isoforms cPGES and mPGES-2 are constitutively expressed (Sampey et al., 2005). mPGES-1 is induced by inflammatory stimuli and the expression levels are increased in the inflamed mucosa of patients with IBD (Subbaramaiah et al., 2004). PGES isomers are functionally coupled to respective COX isomers. For instance, mPGES-1 is preferably coupled to COX-2 and thus produces PGE_2_ in a COX-2-dependent way (Murakami et al., 2000). Thus, the unaffected COX-2 gene expression levels might explain why in turn no upregulation of mPGES-1 was observed. Colorectal carcinoma is (as of yet) the only tissue in which an increased mPGES-2 expression was reported (Murakami et al., 2003).

We have also observed that *in vivo,* the effect of the CaSR modulators depend on an inflamed environment, as treatment with the CaSR modulators affected certain PGE_2_ pathway genes only in the colons of mice with DSS-induced colitis, but not in the colons of mice without colitis, indicating a selective action of the CaSR in an inflamed environment. This fits also well with our experiment of inducing exogenous inflammation in the HT29 cells using rising levels of IL-1β. The CaSR expressing HT29^CaSR−GFP^ cells reacted with much stronger upregulation of IL-8 gene expression to IL-1β than the control HT29^GFP^ cells, indicating that the presence of the CaSR alone already may increase the cells’ susceptibility, or “prime” them, towards inflammatory stimuli, *e.g.*, as suggested by the observation that NPS *R*-568 upregulated IL-1R1 expression in HT29^CaSR−GFP^ cells [RNASeq data from (Iamartino et al., 2020)].

In our previous study (Elajnaf et al., 2019) we found that the calcilytic NPS 2143 reduced the colitis symptoms in mice. In our current study, we could show that the anti-inflammatory effect of NPS 2143 was, at least partially, mediated via the upregulation of 15-PGDH. An antitumor potential of the PGE_2_-degrading enzyme 15-PGDH was already demonstrated as decreased 15-PGDH levels were shown to correlate with colitis-associated colon cancer development (Choi et al., 2014). Contrary to this, an inhibition of 15-PGDH was shown to promote tissue regeneration and reduced inflammatory cytokines in the colons of a DSS-induced colitis mouse model (Zhang et al., 2015b; Hu et al., 2022). Calcilytics, such as NPS 2143, have been shown to reduce inflammation, and disease-associated symptoms in asthma and pulmonary fibrosis, and are now in development for its therapy (Yarova et al., 2015; Yarova et al., 2018; Yarova et al., 2021). Moreover, NPS 2143 has been shown to ameliorate β-amyloid-induced cognitive deficits, which is partially mediated via the cytosolic phospholipase A_2_ and PGE_2_ pathway (Feng et al., 2020). In agreement with the before-mentioned findings, we suggest that calcilytics might also show potential as novel substances for the prevention of colitis and colitis-associated colon cancer, providing a safer alternative to non-steroidal anti-inflammatory drugs (NSAIDs) with less side-effects, as the use of NSAIDs to inhibit COX-2 was found to exacerbate IBD (Meyer et al., 2006; Long et al., 2016).

This study sheds further light on the pro-inflammatory actions of the CaSR. Despite the differences between the more artificial *in vitro* model system and the *in vivo* results, it is clear that activating the CaSR in the colon modulates the PGE_2_ pathway as one of the underlying mechanisms for the pro-inflammatory response following CaSR activation. Our results are in line with other studies that demonstrated increased PGE_2_ production after CaSR stimulation in neurons, dental pulp cells, and fibroblasts from jaw cysts and gingiva (Ogata et al., 2006; Feng et al., 2020; An et al., 2022). Apart from the PGE_2_ pathway, the CaSR also promotes inflammation via activation of the NLRP3 inflammasome in monocytes and adipocytes (Rossol et al., 2012; D'Espessailles et al., 2018; Jäger et al., 2020) as well as the NF-κB pathway in peripheral blood (Li et al., 2013). It is evident that the NLRP3 inflammasome (Bauer et al., 2010; Zhen and Zhang, 2019) and NF-κB pathway (Atreya et al., 2008) may contribute to intestinal inflammation, however the role of the CaSR in this context is yet to be determined.

## 5 Conclusion

Here, we demonstrate for the first time that activation of the CaSR induces the PGE_2_ pathway in CaSR-transfected colon cancer cells and in inflamed mouse colons. CaSR-mediated inflammatory responses in the colon may therefore be, at least in part, mediated through the PGE_2_ pathway.

## Data Availability

The original contributions presented in the study are included in the article/Supplementary Material, further inquiries can be directed to the corresponding author.

## Abbreviations

CaSR, Calcium-sensing receptor; IBD, Inflammatory bowel disease; PGE_2_, Prostaglandin E_2_; COX, Cyclooxygenase; PGES, Prostaglandin E synthase; 15-PGDH, 15-Hydroxyprostaglandin dehydrogenase; DSS, Dextran sulphate sodium.
