# Supplementary figures and images for: The calcium-sensing receptor modulates the prostaglandin E2 pathway in intestinal inflammation
Source: Front Pharmacol. 2023 Apr 20;14:1151144. doi: 10.3389/fphar.2023.1151144 (PMC10157649; doi:10.3389/fphar.2023.1151144)

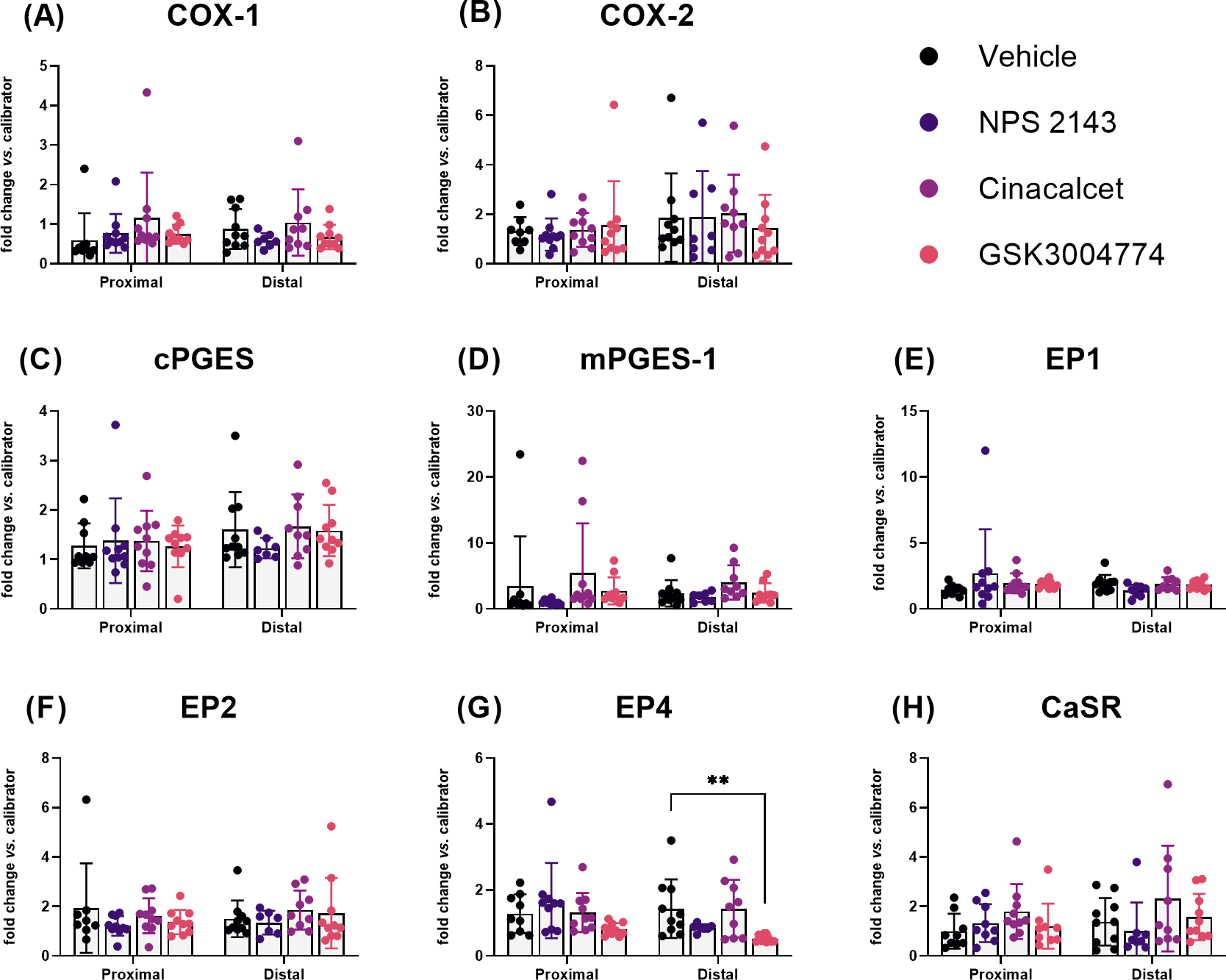

Supplement: Supplementary file 1 [file Image6.TIF]

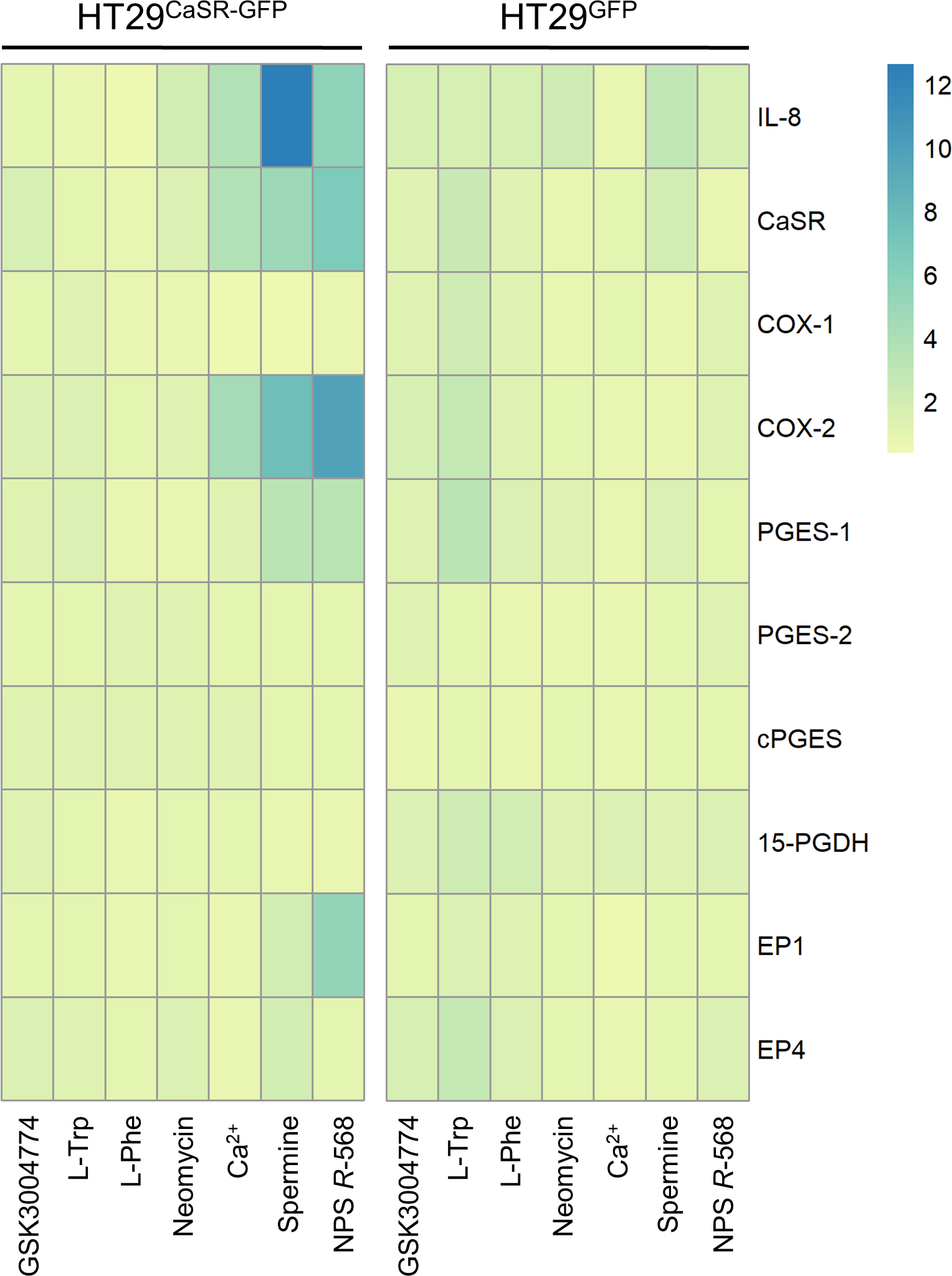

Supplement: Supplementary file 3 [file Image3.TIF]

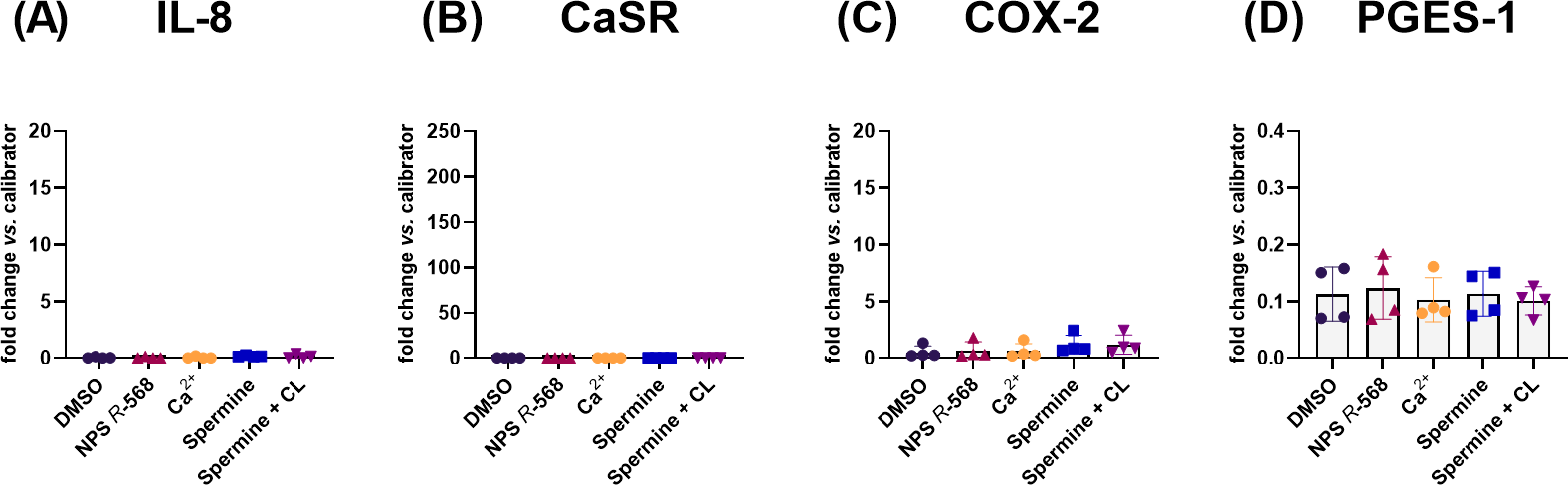

Supplement: Supplementary file 4 [file Image4.TIF]

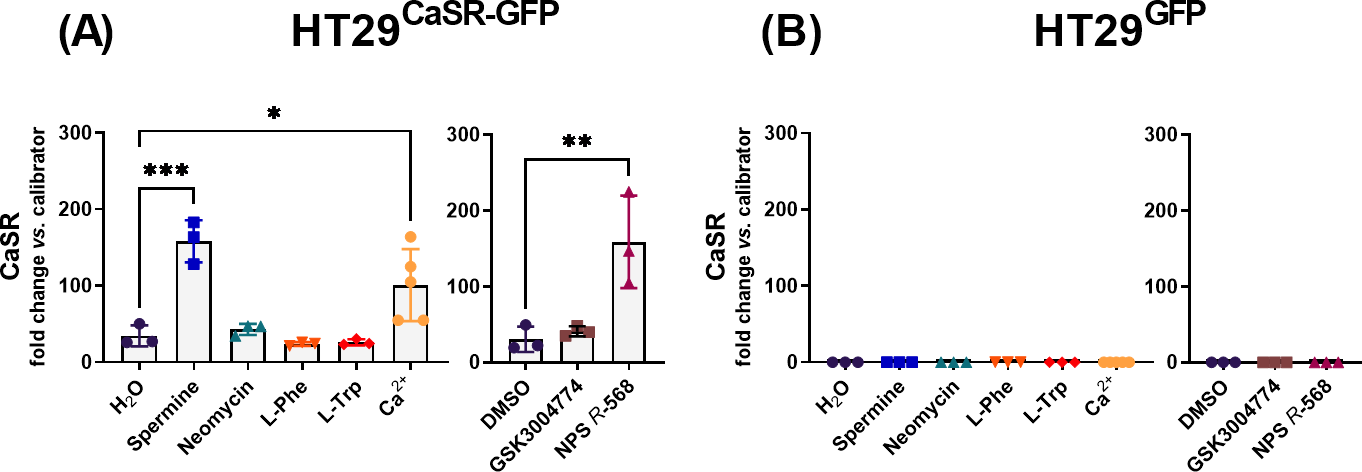

Supplement: Supplementary file 5 [file Image2.TIF]

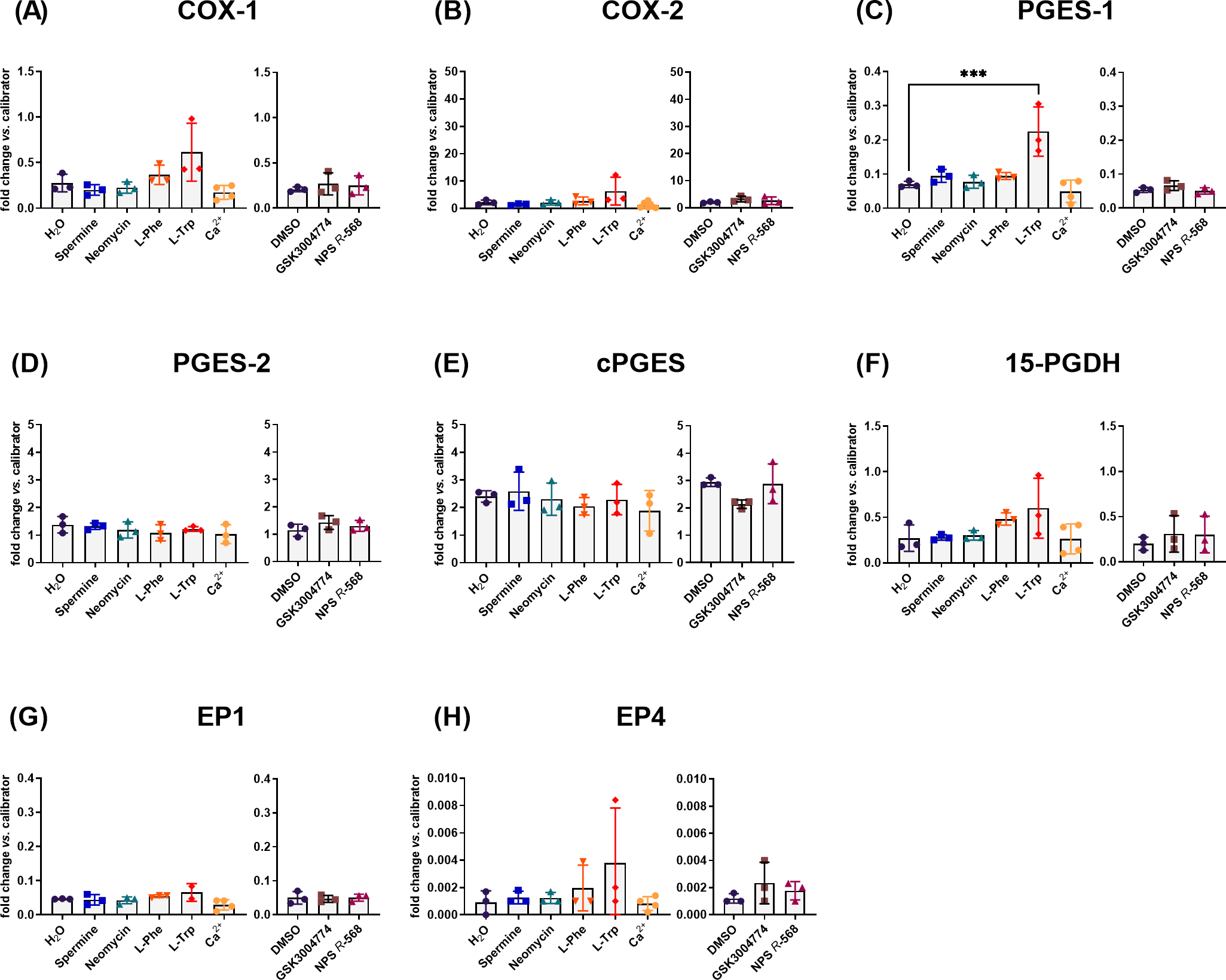

Supplement: Supplementary file 6 [file Image1.TIF]

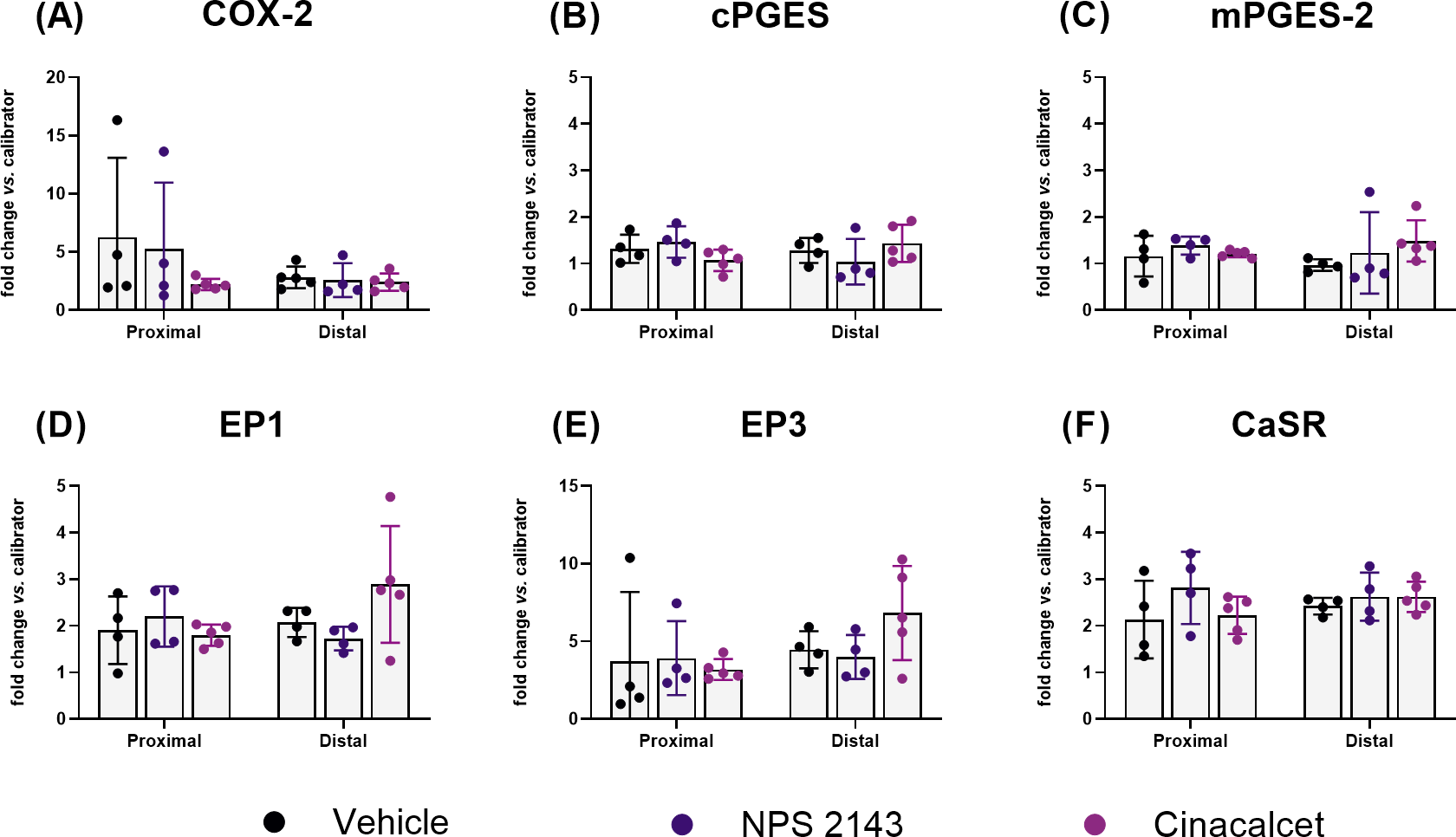

Supplement: Supplementary file 7 [file Image7.TIF]

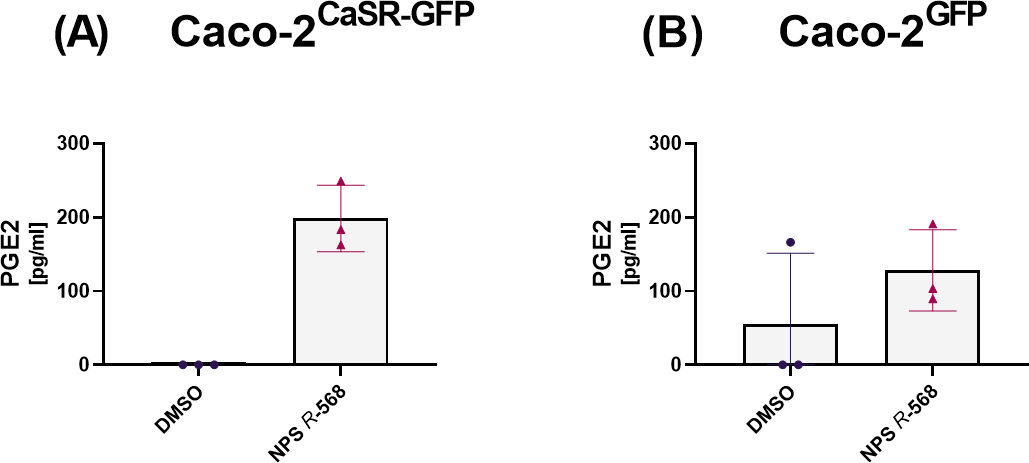

Supplement: Supplementary file 11 [file Image5.TIF]
