# Supplementary material for: The calcium-sensing receptor modulates the prostaglandin E2 pathway in intestinal inflammation
Source: Front Pharmacol. 2023 Apr 20;14:1151144. doi: 10.3389/fphar.2023.1151144 (PMC10157649; doi:10.3389/fphar.2023.1151144)
Supplement: Supplementary file 2 [file Table1.DOCX]

Table S1. RT-qPCR primer list. F, Forward; R, Reverse.

| Gene name |  | Sequence (5'→3') |
| --- | --- | --- |
| Human primers |  |  |
| RPLP0 | F: | TGGTCATCCAGCAGGTGTTCGA |
|  | R: | GCAGCAGCTGGCACCTTATTG |
| B2m Ex 2-4 | F: | GATGAGTATGCCTGCCGTGTG |
|  | R: | CAATCCAAATGCGGCATCT |
| IL8 | F: | CTTGGCAGCCTTCCTGATTT |
|  | R: | TTCTTTAGCACTCCTTGGCAAAA |
| CaSR Ex 5-6 | F: | GCCAAGAAGGGAGAAAGAC |
|  | R: | CACACTCAAAGCAGCAGG |
| COX-1 | F: | TTGAATGAGTACCGCAAGAGG |
|  | R: | GAAGCAGTCCAGGGTAGAAC |
| COX-2 | F: | CAAGACAGATCATAAGCGAGGG |
|  | R: | GTCTAGCCAGAGTTTCACCG |
| cPGES | F: | ATG CAG CCT GCT TCT GCA AA |
|  | R: | CCT TAC TCC AGA TCT GGC AT |
| PGES-1 | F: | GCTGGTCATCAAGATGTACG |
|  | R: | GTCGCTCCTGCAATACTG |
| PGES-2 | F: | GCA GCT GAC CCT GTA CCA GT |
|  | R: | CTC GCG GAC AAT GTA GTC AA |
| 15-PGDH | F: | TGCTTCAAAGCATGGCATG |
|  | R: | AACAAGCCTGGACAAATGG |
| EP1 | F: | GGCCAGCTTGTCGGT |
|  | R: | GCCACCAAGACCAGC |
| EP2 | F: | GCTATCATGACCATCACCT |
|  | R: | CCTAAGAGCTTGGAGGTC |
| EP3 | F: | TGGTCTCCGCTCCTGATAATG |
|  | R: | TGTGTCTTGCAGTGCTCAACTG |
| EP4 | F: | TGCTCATCTGCTCCATGG |
|  | R: | TTACTGACTTCTCGCTCCA |
| Mouse primers |  |  |
| β-actin | F: | TGACGGGGTCACCCACACTGTGCCCATCTA |
|  | R: | CTAGAAGCATTTGCGGTGGACGATGGAGGG |
| Eef1β2 | F: | TACATTGAGGGGTACGTGCCAT |
|  | R: | GGTGGACCAGAAACTGCTTCA |
| CaSR | F: | TGGTGAGACAGATGCGAGT |
|  | R: | GCCAGGAACTCAATCTCCTT |
| COX-1 | F: | ATGAGTCGAAGGAGTCTCTCG |
|  | R: | GCACGGATAGTAACAACAGGGA |
| COX-2 | F: | TGAGCAACTATTCCAAACCAGC |
|  | R: | GCACGTAGTCTTCGATCACTATC |
| cPGES | F: | GGTAGAGACCGCCGGAGT |
|  | R: | TCGTACCACTTTGCAGAAGCA |
| mPGES-1 | F: | AGCACACTGCTGGTCATCAA |
|  | R: | CTCCACATCTGGGTCACTCC |
| mPGES-2 | F: | GCTGGGGCTGTACCACAC |
|  | R: | GATTCACCTCCACCACCTGA |
| 15-PGDH | F: | GTTCGTCCAGTGTGATGTGG |
|  | R: | CCTTCACCTCCGTTTTGCTT |
| EP1 | F: | GGGCTTAACCTGAGCCTAGC |
|  | R: | GTGATGTGCCATTATCGCTG |
| EP2 | F: | TCCCTAAAGGAAAAGTGGGACC |
|  | R: | GAGCGCATTAACCTCAGGACC |
| EP3 | F: | CCGGAGCACTCTGCTGAAG |
|  | R: | CCCCACTAAGTCGGTGAGC |
| EP4 | F: | ACCATTCCTAGATCGAACCGT |
|  | R: | CACCACCCCGAAGATGAACAT |
