# Supplementary material for: The calcium-sensing receptor modulates the prostaglandin E2 pathway in intestinal inflammation
Source: Front Pharmacol. 2023 Apr 20;14:1151144. doi: 10.3389/fphar.2023.1151144 (PMC10157649; doi:10.3389/fphar.2023.1151144)
Supplement: Supplementary file 8 [file Table2.DOCX]

Table S2. RT-qPCR results of targeted genes in HT29^CaSR-GFP^ and HT29^GFP^ cells treated under different conditions. Values are depicted as mean (fold change vs. calibrator) ± standard deviation (SD). Statistical analysis was performed with one-way ANOVA with Dunnett’s post-hoc test *vs*. vehicle (H_2_O or DMSO), not significant (ns), * *p* < 0.05, ** *p* < 0.01, *** *p* < 0.001.

|  |  | **HT29^CaSR-GFP^** | | |  | **HT29^GFP^** | | |  |  | **HT29^CaSR-GFP^** | | |  | **HT29^GFP^** | | |
| --- | --- | --- | --- | --- | --- | --- | --- | --- | --- | --- | --- | --- | --- | --- | --- | --- | --- |
| **Gene** | **Treatment** | **Mean (± SD)** | ***P* value** |  |  | **Mean (± SD)** | ***P* value** |  | **Gene** | **Treatment** | **Mean (± SD)** | ***P* value** |  |  | **Mean (± SD)** | ***P* value** |  |
| IL8 | H_2_O | 2.67 ± 1.25 |  |  |  | 1.302 ± 0.607 |  |  | cPGES | H_2_O | 1.461 ± 0.348 |  |  |  | 2.400 ± 0.206 |  |  |
|  | Spermine | 29.64 ± 12.88 | <0.001 | *** |  | 3.240 ± 0.394 | 0.065 | ns |  | Spermine | 1.492 ± 0.234 | 0.999 | ns |  | 2.588 ± 0.693 | 0.991 | ns |
|  | Neomycin | 3.84 ± 1.54 | >0.999 | ns |  | 3.111 ± 1.740 | 0.089 | ns |  | Neomycin | 1.742 ± 0.276 | 0.734 | ns |  | 2.303 ± 0.583 | 0.999 | ns |
|  | L-Phe | 1.18 ± 0.34 | >0.999 | ns |  | 2.014 ± 0.401 | 0.775 | ns |  | L-Phe | 1.821 ± 0.257 | 0.541 | ns |  | 2.046 ± 0.318 | 0.889 | ns |
|  | L-Trp | 1.32 ± 0.40 | >0.999 | ns |  | 2.317 ± 1.071 | 0.501 | ns |  | L-Trp | 1.588 ± 0.096 | 0.983 | ns |  | 2.287 ± 0.550 | 0.999 | ns |
|  | L-Phe 5 mM | 0.92 ± 0.21 | NA | NA |  |  |  |  |  |  |  |  |  |  |  |  |  |
|  | L-Phe 10 mM | 0.59 ± 0.11 | NA | NA |  |  |  |  |  |  |  |  |  |  |  |  |  |
|  | L-Trp 5 mM | 1.25 ± 0.07 | NA | NA |  |  |  |  |  |  |  |  |  |  |  |  |  |
|  | L-Trp 10 mM | 2.60 ± 0.36 | NA | NA |  |  |  |  |  |  |  |  |  |  |  |  |  |
|  | Ca^2+^ | 7.07 ± 2.81 | 0.67 | ns |  | 0.791 ± 0.544 | 0.883 | ns |  | Ca^2+^ | 2.042 ± 0.551 | 0.165 | ns |  | 1.885 ± 0.732 | 0.678 | ns |
|  | DMSO | 2.27 ± 0.79 |  |  |  | 0.867 ± 0.192 |  |  |  | DMSO | 2.071 ± 0.053 |  |  |  | 2.939 ± 0.155 |  |  |
|  | GSK3004774 | 2.08 ± 0.33 | 0.996 | ns |  | 1.510 ± 0.875 | 0.500 | ns |  | GSK3004774 | 2.355 ± 0.653 | 0.691 | ns |  | 2.137 ± 0.154 | 0.113 | ns |
|  | NPS R-568 | 10.87 ± 5.62 | 0.032 | * |  | 1.499 ± 0.908 | 0.510 | ns |  | NPS R-568 | 1.936 ± 0.470 | 0.914 | ns |  | 2.880 ± 0.728 | 0.980 | ns |
| CaSR | H_2_O | 34.43 ± 43.82 |  |  |  | 0.0037 ± 0.0029 |  |  | 15-PGDH | H_2_O | 0.373 ± 0.083 |  |  |  | 0.273 ± 0.145 |  |  |
|  | Spermine | 158.3 ± 27.6 | <0.001 | *** |  | 0.0103 ± 0.014 | 0.611 | ns |  | Spermine | 0.277 ± 0.122 | 0.855 | ns |  | 0.280 ± 0.030 | >0.999 | ns |
|  | Neomycin | 42.98 ± 7.45 | >0.999 | ns |  | 0.0030 ± 0.0026 | 0.999 | ns |  | Neomycin | 0.336 ± 0.174 | 0.996 | ns |  | 0.302 ± 0.052 | 0.999 | ns |
|  | L-Phe | 23.94 ± 2.42 | 0.990 | ns |  | 0.0053 ± 0.0049 | 0.997 | ns |  | L-Phe | 0.314 ± 0.115 | 0.975 | ns |  | 0.482 ± 0.068 | 0.422 | ns |
|  | L-Trp | 26.23 ± 3.97 | >0.999 | ns |  | 0.0093 ± 0.0087 | 0.734 | ns |  | L-Trp | 0.340 ± 0.104 | 0.998 | ns |  | 0.600 ± 0.329 | 0.108 | ns |
|  | Ca^2+^ | 100.9 ± 46.9 | 0.020 | * |  | 0.0024 ± 0.0015 | 0.999 | ns |  | Ca^2+^ | 0.310 ± 0.174 | 0.957 | ns |  | 0.263 ± 0.164 | 0.999 | ns |
|  | DMSO | 30.42 ± 16.68 |  |  |  | 0.0040 ± 0.0030 |  |  |  | DMSO | 0.328 ± 0.112 |  |  |  | 0.201 ± 0.072 |  |  |
|  | GSK3004774 | 41.1 ± 6.6 | 0.913 | ns |  | 0.0043 ± 0.0025 | 0.989 | ns |  | GSK3004774 | 0.363 ± 0.112 | 0.929 | ns |  | 0.309 ± 0.202 | 0.671 | ns |
|  | NPS R-568 | 158.8 ± 61.0 | 0.009 | ** |  | 0.0037 ± 0.0040 | 0.989 | ns |  | NPS R-568 | 0.244 ± 0.172 | 0.683 | ns |  | 0.302 ± 0.203 | 0.703 | ns |
| COX-1 | H_2_O | 0.766 ± 0.137 |  |  |  | 0.276 ± 0.096 |  |  | EP1 | H_2_O | 0.069 ± 0.019 |  |  |  | 0.046 ± 0.001 |  |  |
|  | Spermine | 0.391 ± 0.030 | 0.006 | ** |  | 0.201 ± 0.058 | 0.947 | ns |  | Spermine | 0.132 ± 0.062 | 0.200 | ns |  | 0.044 ± 0.015 | 0.999 | ns |
|  | Neomycin | 0.566 ± 0.177 | 0.179 | ns |  | 0.226 ± 0.062 | 0.991 | ns |  | Neomycin | 0.062 ± 0.027 | >0.999 | ns |  | 0.043 ± 0.010 | 0.995 | ns |
|  | L-Phe | 0.584 ± 0.111 | 0.242 | ns |  | 0.367 ± 0.107 | 0.991 | ns |  | L-Phe | 0.050 ± 0.020 | 0.940 | ns |  | 0.055 ± 0.004 | 0.904 | ns |
|  | L-Trp | 0.862 ± 0.075 | 0.760 | ns |  | 0.615 ± 0.318 | 0.052 | ns |  | L-Trp | 0.066 ± 0.026 | >0.999 | ns |  | 0.065 ± 0.026 | 0.406 | ns |
|  | Ca^2+^ | 0.237 ± 0.101 | 0.0002 | *** |  | 0.174 ± 0.077 | 0.816 | ns |  | Ca^2+^ | 0.084 ± 0.043 | 0.970 | ns |  | 0.030 ± 0.015 | 0.349 | ns |
|  | DMSO | 0.565 ± 0.101 |  |  |  | 0.207 ± 0.026 |  |  |  | DMSO | 0.055 ± 0.023 |  |  |  | 0.050 ± 0.019 |  |  |
|  | GSK3004774 | 0.550 ± 0.086 | 0.983 | ns |  | 0.267 ± 0.122 | 0.983 | ns |  | GSK3004774 | 0.052 ± 0.002 | 0.995 | ns |  | 0.047 ± 0.010 | 0.946 | ns |
|  | NPS R-568 | 0.404 ± 0.146 | 0.225 | ns |  | 0.251 ± 0.106 | 0.225 | ns |  | NPS R-568 | 0.269 ± 0.072 | 0.0018 | ** |  | 0.050 ± 0.010 | 0.999 | ns |
| COX-2 | H_2_O | 3.01 ± 0.17 |  |  |  | 2.21 ± 0.85 |  |  | EP2 | H_2_O | ND |  |  |  | ND |  |  |
|  | Spermine | 22.98 ± 7.31 | 0.005 | ** |  | 1.47 ± 0.33 | 0.988 | ns |  | Spermine | ND |  |  |  | ND |  |  |
|  | Neomycin | 3.83 ± 1.19 | >0.999 | ns |  | 2.18 ± 0.95 | >0.999 | ns |  | Neomycin | ND |  |  |  | ND |  |  |
|  | L-Phe | 2.69 ± 0.38 | >0.999 | ns |  | 2.76 ± 1.41 | 0.997 | ns |  | L-Phe | ND |  |  |  | ND |  |  |
|  | L-Trp | 4.62 ± 1.23 | >0.999 | ns |  | 6.33 ± 5.08 | 0.100 | ns |  | L-Trp | ND |  |  |  | ND |  |  |
|  | Ca^2+^ | 12.50 ± 9.80 | 0.160 | ns |  | 1.13 ± 1.08 | 0.907 | ns |  | Ca^2+^ | ND |  |  |  | ND |  |  |
|  | DMSO | 3.15 ± 0.70 |  |  |  | 2.00 ± 0.29 |  |  |  | DMSO | ND |  |  |  | ND |  |  |
|  | GSK3004774 | 4.51 ± 0.66 | 0.887 | ns |  | 3.22 ± 1.02 | 0.313 | ns |  | GSK3004774 | ND |  |  |  | ND |  |  |
|  | NPS R-568 | 29.44 ± 6.92 | 0.0004 | *** |  | 2.62 ± 1.41 | 0.693 | ns |  | NPS R-568 | ND |  |  |  | ND |  |  |
| PGES-1 | H_2_O | 0.113 ± 0.039 |  |  |  | 0.070 ± 0.094 |  |  | EP3 | H_2_O | ND |  |  |  | ND |  |  |
|  | Spermine | 0.326 ± 0.050 | 0.0002 | *** |  | 0.095 ± 0.019 | 0.834 | ns |  | Spermine | ND |  |  |  | ND |  |  |
|  | Neomycin | 0.082 ± 0.008 | 0.832 | ns |  | 0.077 ± 0.019 | 0.998 | ns |  | Neomycin | ND |  |  |  | ND |  |  |
|  | L-Phe | 0.074 ± 0.002 | 0.703 | ns |  | 0.095 ± 0.010 | 0.835 | ns |  | L-Phe | ND |  |  |  | ND |  |  |
|  | L-Trp | 0.175 ± 0.048 | 0.309 | ns |  | 0.225 ± 0.072 | 0.0004 | *** |  | L-Trp | ND |  |  |  | ND |  |  |
|  | Ca^2+^ | 0.118 ± 0.060 | 0.999 | ns |  | 0.050 ± 0.033 | 0.901 | ns |  | Ca^2+^ | ND |  |  |  | ND |  |  |
|  | DMSO | 0.072 ± 0.009 |  |  |  | 0.054 ± 0.007 |  |  |  | DMSO | ND |  |  |  | ND |  |  |
|  | GSK3004774 | 0.078 ± 0.009 | 0.966 | ns |  | 0.066 ± 0.014 | 0.333 | ns |  | GSK3004774 | ND |  |  |  | ND |  |  |
|  | NPS R-568 | 0.236 ± 0.054 | 0.0014 | ** |  | 0.051 ± 0.009 | 0.923 | ns |  | NPS R-568 | ND |  |  |  | ND |  |  |
| PGES-2 | H_2_O | 1.189 ± 0.370 |  |  |  | 1.378 ± 0.298 |  |  | EP4 | H_2_O | 0.0010 ± 0.00001 |  |  |  | 0.0009 ± 0.0009 |  |  |
|  | Spermine | 1.130 ± 0.352 | 0.999 | ns |  | 1.323 ± 0.114 | 0.999 | ns |  | Spermine | 0.0021 ± 0.0002 | 0.0493 | *** |  | 0.0013 ± 0.0005 | 0.999 | ns |
|  | Neomycin | 1.515 ± 0.294 | 0.584 | ns |  | 1.188 ± 0.290 | 0.827 | ns |  | Neomycin | 0.0016 ± 0.0010 | 0.520 | ns |  | 0.0012 ± 0.0004 | 0.999 | ns |
|  | L-Phe | 1.331 ± 0.213 | 0.968 | ns |  | 1.085 ± 0.290 | 0.516 | ns |  | L-Phe | 0.0011 ± 0.0001 | 0.999 | ns |  | 0.0020 ± 0.0017 | 0.910 | ns |
|  | L-Trp | 1.190 ± 0.107 | >0.999 | ns |  | 1.222 ± 0.086 | 0.908 | ns |  | L-Trp | 0.0012 ± 0.0003 | 0.996 | ns |  | 0.0038 ± 0.0040 | 0.218 | ns |
|  | Ca^2+^ | 1.370 ± 0.405 | 0.919 | ns |  | 0.104 ± 0.340 | 0.385 | ns |  | Ca^2+^ | 0.0010 ± 0.0005 | 0.999 | ns |  | 0.0008 ± 0.0005 | >0.999 | ns |
|  | DMSO | 2.133 ± 1.317 |  |  |  | 1.143 ± 0.223 |  |  |  | DMSO | 0.0019 ± 0.0001 |  |  |  | 0.0012 ± 0.0003 |  |  |
|  | GSK3004774 | 1.496 ± 0.316 | 0.568 | ns |  | 1.430 ± 0.253 | 0.278 | ns |  | GSK3004774 | 0.0031 ± 0.0010 | 0.402 | ns |  | 0.0023 ± 0.0015 | 0.335 | ns |
|  | NPS R-568 | 1.709 ± 0.448 | 0.763 | ns |  | 1.303 ± 0.199 | 0.616 | ns |  | NPS R-568 | 0.0019 ± 0.0016 | 0.996 | ns |  | 0.0018 ± 0.0007 | 0.718 | ns |
