# Supplementary material for: The calcium-sensing receptor modulates the prostaglandin E2 pathway in intestinal inflammation
Source: Front Pharmacol. 2023 Apr 20;14:1151144. doi: 10.3389/fphar.2023.1151144 (PMC10157649; doi:10.3389/fphar.2023.1151144)
Supplement: Supplementary file 9 [file Table3.DOCX]

Table S3. RT-qPCR results of targeted genes in Caco-2^CaSR-GFP^ and Caco-2^GFP^ cells treated under different conditions. Values are depicted as mean (fold change vs. calibrator) ± standard deviation (SD). Statistical analysis was performed with one-way ANOVA with Dunnett’s post-hoc test *vs*. vehicle (H_2_O or DMSO), not significant (ns), ** *p* < 0.01, **** *p* < 0.0001.

|  |  | **Caco-2^CaSR-GFP^** | | |  | **Caco-2^GFP^** | | |
| --- | --- | --- | --- | --- | --- | --- | --- | --- |
| **Gene** | **Treatment** | **Mean (± SD)** | ***P* value** |  |  | **Mean (± SD)** | ***P* value** |  |
| IL8 | DMSO | 0.03 ± 0.03 |  |  |  | 0.042 ± 0.052 |  |  |
|  | Spermine | 10.27 ± 5.82 | <0.0001 | **** |  | 0.159 ± 0.066 | 0.303 | ns |
|  | Ca^2+^ | 0.95 ± 0.51 | 0.950 | ns |  | 0.059 ± 0.085 | 0.998 | ns |
|  | NPS R-568 | 2.32 ± 0.68 | 0.412 | ns |  | 0.045 ± 0.063 | >0.999 | ns |
| CaSR | DMSO | 26.07 ± 3.36 |  |  |  | 0.010 ± 0.008 |  |  |
|  | Spermine | 140.0 ± 51.6 | <0.0001 | **** |  | 0.014 ± 0.010 | 0.998 | ns |
|  | Ca^2+^ | 76.25 ± 27.36 | 0.103 | ns |  | 0.014 ± 0.006 | 0.998 | ns |
|  | NPS R-568 | 112.9 ± 31.98 | 0.0016 | ** |  | 0.030 ± 0.039 | 0.541 | ns |
| COX-2 | DMSO | 1.21 ± 0.65 |  |  |  | 0.512 ± 0.541 |  |  |
|  | Spermine | 12.93 ± 2.07 | <0.0001 | **** |  | 1.193 ± 0.833 | 0.618 | ns |
|  | Ca^2+^ | 5.58 ± 1.67 | 0.0077 | ** |  | 0.6009 ± 0.680 | 0.999 | ns |
|  | NPS R-568 | 8.65 ± 2.81 | <0.0001 | **** |  | 0.6534 ± 0.766 | 0.998 | ns |
| PGES | DMSO | 0.105 ± 0.027 |  |  |  | 0.113 ± 0.048 |  |  |
|  | Spermine | 0.107 ± 0.028 | 0.999 | ns |  | 0.114 ± 0.039 | >0.999 | ns |
|  | Ca^2+^ | 0.097 ± 0.025 | 0.953 | ns |  | 0.103 ± 0.039 | 0.992 | ns |
|  | NPS R-568 | 0.092 ± 0.023 | 0.830 | ns |  | 0.124 ± 0.055 | 0.989 | ns |
