# Supplementary material for: The calcium-sensing receptor modulates the prostaglandin E2 pathway in intestinal inflammation
Source: Front Pharmacol. 2023 Apr 20;14:1151144. doi: 10.3389/fphar.2023.1151144 (PMC10157649; doi:10.3389/fphar.2023.1151144)
Supplement: Supplementary file 10 [file Table4.DOCX]

Table S4. RT-qPCR results of targeted genes in the proximal and distal colons of mice with DSS-induced colitis (inflamed colon) or no colitis (non-inflamed colon) treated with different CaSR modulators. Values are depicted as mean (fold change vs. calibrator) ± standard deviation (SD). Statistical analysis was performed with one-way ANOVA with Dunnett’s post-hoc test *vs*. vehicle (H_2_O or DMSO), not significant (ns), * *p* < 0.05, ** *p* < 0.01, *** *p* < 0.001, **** *p* < 0.0001.

|  |  | **Inflamed** | | | | | | |  | **Non-inflamed** | | | | | | |
| --- | --- | --- | --- | --- | --- | --- | --- | --- | --- | --- | --- | --- | --- | --- | --- | --- |
|  |  | **Proximal colon** | | |  | **Distal colon** | | |  | **Proximal colon** | | |  | **Distal colon** | | |
| **Genes** | **Treatment** | **Mean (± SD)** | ***P* value** |  |  | **Mean (± SD)** | ***P* value** |  |  | **Mean (± SD)** | ***P* value** |  |  | **Mean (± SD)** | ***P* value** |  |
| COX-1 | Vehicle | 0.592 ± 0.683 |  |  |  | 0.878 ± 0.505 |  |  |  |  |  |  |  |  |  |  |
|  | NPS 2143 | 0.767 ± 0.489 | 0.911 | ns |  | 0.593 ± 0.182 | 0.576 | ns |  |  |  |  |  |  |  |  |
|  | Cinacalcet | 1.155 ± 1.148 | 0.228 | ns |  | 1.041 ± 0.841 | 0.848 | ns |  |  |  |  |  |  |  |  |
|  | GSK3004774 | 0.746 ± 0.239 | 0.936 | ns |  | 0.677 ± 0.307 | 0.740 | ns |  |  |  |  |  |  |  |  |
| COX-2 | Vehicle | 1.314 ± 0.570 |  |  |  | 1.865 ± 1.791 |  |  |  | 6.28 ± 6.82 |  |  |  | 2.82 ± 0.93 |  |  |
|  | NPS 2143 | 1.175 ± 0.653 | 0.984 | ns |  | 1.891 ± 1.859 | >0.999 | ns |  | 5.26 ± 5.70 | 0.938 | ns |  | 2.58 ± 1.46 | 0.920 | ns |
|  | Cinacalcet | 1.372 ± 0.688 | 0.999 | ns |  | 2.028 ± 1.573 | 0.993 | ns |  | 2.20 ± 0.48 | 0.384 | ns |  | 2.42 ± 0.74 | 0.774 | ns |
|  | GSK3004774 | 1.567 ± 1.771 | 0.918 | ns |  | 1.440 ± 1.346 | 0.893 | ns |  |  |  |  |  |  |  |  |
| cPGES | Vehicle | 1.277 ± 0.456 |  |  |  | 1.603 ± 0.760 |  |  |  | 1.32 ± 0.31 |  |  |  | 1.28 ± 0.27 |  |  |
|  | NPS 2143 | 1.378 ± 0.857 | 0.968 | ns |  | 1.230 ± 0.209 | 0.455 | ns |  | 1.47 ± 0.34 | 0.704 | ns |  | 1.04 ± 0.49 | 0.613 | ns |
|  | Cinacalcet | 1.375 ± 0.609 | 0.971 | ns |  | 1.670 ± 0.647 | 0.990 | ns |  | 1.07 ± 0.23 | 0.366 | ns |  | 1.43 ± 0.40 | 0.795 | ns |
|  | GSK3004774 | 1.264 ± 0.422 | >0.999 | ns |  | 1.585 ± 0.519 | 0.999 | ns |  |  |  |  |  |  |  |  |
| mPGES1 | Vehicle | 3.477 ± 7.516 |  |  |  | 2.357 ± 2.014 |  |  |  |  |  |  |  |  |  |  |
|  | NPS 2143 | 0.988 ± 0.358 | 0.614 | ns |  | 1.771 ± 0.724 | 0.871 | ns |  |  |  |  |  |  |  |  |
|  | Cinacalcet | 5.446 ± 7.534 | 0.757 | ns |  | 4.004 ± 2.597 | 0.166 | ns |  |  |  |  |  |  |  |  |
|  | GSK3004774 | 2.742 ± 2.056 | 0.981 | ns |  | 2.417 ± 1.465 | 0.999 | ns |  |  |  |  |  |  |  |  |
| mPGES2 | Vehicle | 0.801 ± 0.342 |  |  |  | 0.977 ± 0.514 |  |  |  | 1.16 ± 0.44 |  |  |  | 0.970 ± 0.125 |  |  |
|  | NPS 2143 | 1.708 ± 1.124 | 0.171 | ns |  | 0.650 ± 0.281 | 0.640 | ns |  | 1.39 ± 0.19 | 0.402 | ns |  | 1.233 ± 0.8755 | 0.736 | ns |
|  | Cinacalcet | 3.213 ± 1.640 | <0.0001 | **** |  | 2.374 ± 1.109 | 0.0003 | *** |  | 1.21 ± 0.08 | 0.944 | ns |  | 1.488 ± 0.4440 | 0.321 | ns |
|  | GSK3004774 | 2.704 ± 0.592 | 0.0012 | ** |  | 1.781 ± 0.589 | 0.0378 | * |  |  |  |  |  |  |  |  |
| 15-PGDH | Vehicle | 2.294 ± 2.087 |  |  |  | 1.001 ± 0.631 |  |  |  |  |  |  |  |  |  |  |
|  | NPS 2143 | 4.129 ± 2.148 | 0.050 | ns |  | 0.680 ± 0.556 | 0.639 | ns |  |  |  |  |  |  |  |  |
|  | Cinacalcet | 1.467 ± 1.110 | 0.552 | ns |  | 0.656 ± 0.996 | 0.535 | ns |  |  |  |  |  |  |  |  |
|  | GSK3004774 | 1.612 ± 0.8006 | 0.685 | ns |  | 0.237 ± 0.155 | 0.0357 | * |  |  |  |  |  |  |  |  |
| EP1 | Vehicle | 1.446 ± 0.400 |  |  |  | 1.929 ± 0.642 |  |  |  | 1.905 ± 0.725 |  |  |  | 2.073 ± 0.3126 |  |  |
|  | NPS 2143 | 2.688 ± 3.356 | 0.298 | ns |  | 1.381 ± 0.453 | 0.071 | ns |  | 2.200 ± 0.647 | 0.675 | ns |  | 1.728 ± 0.2547 | 0.780 | ns |
|  | Cinacalcet | 1.943 ± 0.750 | 0.868 | ns |  | 1.903 ± 0.507 | 0.999 | ns |  | 1.800 ± 0.228 | 0.942 | ns |  | 2.888 ± 1.254 | 0.280 | ns |
|  | GSK3004774 | 1.876 ± 0.297 | 0.908 | ns |  | 1.827 ± 0.337 | 0.943 | ns |  |  |  |  |  |  |  |  |
| EP2 | Vehicle | 1.933 ± 1.810 |  |  |  | 1.499 ± 0.742 |  |  |  |  |  |  |  |  |  |  |
|  | NPS 2143 | 1.213 ± 0.399 | 0.270 | ns |  | 1.344 ± 0.481 | 0.978 | ns |  |  |  |  |  |  |  |  |
|  | Cinacalcet | 1.621 ± 0.706 | 0.822 | ns |  | 1.853 ± 0.787 | 0.771 | ns |  |  |  |  |  |  |  |  |
|  | GSK3004774 | 1.370 ± 0.494 | 0.456 | ns |  | 1.730 ± 1.429 | 0.913 | ns |  |  |  |  |  |  |  |  |
| EP3 | Vehicle | 1.482 ± 0.582 |  |  |  | 2.822 ± 2.578 |  |  |  | 3.708 ± 4.473 |  |  |  | 4.463 ± 1.194 |  |  |
|  | NPS 2143 | 1.671 ± 0.928 | 0.968 | ns |  | 1.834 ± 1.116 | 0.630 | ns |  | 3.925 ± 2.382 | 0.991 | ns |  | 4.005 ± 1.418 | 0.936 | ns |
|  | Cinacalcet | 3.455 ± 1.223 | 0.0017 | ** |  | 2.399 ± 1.110 | 0.940 | ns |  | 3.190 ± 0.673 | 0.946 | ns |  | 6.826 ± 3.027 | 0.225 | ns |
|  | GSK3004774 | 2.593 ± 1.545 | 0.101 | ns |  | 2.532 ± 2.318 | 0.978 | ns |  |  |  |  |  |  |  |  |
| EP4 | Vehicle | 1.282 ± 0.588 |  |  |  | 1.434 ± 0.849 |  |  |  |  |  |  |  |  |  |  |
|  | NPS 2143 | 1.678 ± 1.139 | 0.486 | ns |  | 0.849 ± 0.114 | 0.185 | ns |  |  |  |  |  |  |  |  |
|  | Cinacalcet | 1.315 ± 0.593 | 0.999 | ns |  | 1.434 ± 0.8714 | >0.999 | ns |  |  |  |  |  |  |  |  |
|  | GSK3004774 | 0.807 ± 0.185 | 0.344 | ns |  | 0.521 ± 0.108 | 0.0097 | ** |  |  |  |  |  |  |  |  |
| CaSR | Vehicle | 0.994 ± 0.706 |  |  |  | 1.376 ± 0.959 |  |  |  | 2.135 ± 0.834 |  |  |  | 2.423 ± 0.178 |  |  |
|  | NPS 2143 | 1.326 ± 0.766 | 0.772 | ns |  | 1.013 ± 1.145 | 0.902 | ns |  | 2.815 ± 0.774 | 0.299 | ns |  | 2.628 ± 0.518 | 0.648 | ns |
|  | Cinacalcet | 1.794 ± 1.109 | 0.147 | ns |  | 2.321 ± 2.136 | 0.324 | ns |  | 2.226 ± 0.401 | 0.970 | ns |  | 2.622 ± 0.324 | 0.635 | ns |
|  | GSK3004774 | 1.203 ± 0.916 | 0.926 | ns |  | 1.572 ± 0.936 | 0.979 | ns |  |  |  |  |  |  |  |  |
