# Supplementary material for: The calcium-sensing receptor modulates the prostaglandin E2 pathway in intestinal inflammation
Source: Front Pharmacol. 2023 Apr 20;14:1151144. doi: 10.3389/fphar.2023.1151144 (PMC10157649; doi:10.3389/fphar.2023.1151144)
Supplement: Supplementary file 12 [file DataSheet1.docx]

Figure S1. Induction of PGE_2_ pathway genes by CaSR ligands in HT29^CaSR-GFP^ cells. Relative gene expression of (A) COX-1, (B) COX-2, (C) PGES-1, (D) PGES-2, (E) cPGES, (F) 15-PGDH, (G) EP1, and (H) EP4 in HT29^GFP^ cells after 4 h treatment with CaSR ligands (left panel: H_2_O as vehicle control, 5 mM spermine, 300 µM neomycin, 1 mM L-Phe, 1 mM L-Trp, and 5 mM Ca^2+^) or CaSR modulators (right panel: 0.1% DMSO as vehicle control,10 µM GSK3004774, and 1 µM NPS *R*-568). Mean ± SD, N = 3-5, one-way ANOVA with Dunnett’s post-hoc test *vs.* vehicle control (H_2_O or 0.1% DMSO), *** *p* < 0.001.

Figure S2. Induction of CaSR by CaSR ligands in HT29^CaSR-GFP^ and HT29^GFP^ cells. Relative gene expression of CaSR in (A) HT29^CaSR-GFP^ and (B) HT29^GFP^ cells after 4 h treatment with CaSR ligands (left panel: H_2_O as vehicle control, 5 mM spermine, 300 µM neomycin, 1 mM L-Phe, 1 mM L-Trp, and 5 mM Ca^2+^) or CaSR modulators (right panel: 0.1% DMSO as vehicle control, 10 µM GSK3004774, and 1 µM NPS *R*-568). Mean ± SD, N = 3-5, one-way ANOVA with Dunnett’s post-hoc test *vs*. vehicle control (H_2_O or 0.1% DMSO), * *p* < 0.05, ** *p* < 0.01, *** *p* < 0.001.

Figure S3. Heatmap showing the expression level of IL8, CaSR, and PGE_2_ pathway genes (rows) after different treatments (columns) in HT29^CaSR-GFP^ and HT29^GFP^ cells. The gradient color scale represents the mean fold change normalized *vs*. vehicle, as indicated in the legend.

Figure S4. Gene expression in Caco-2^GFP^ cells. Relative gene expression of (A) IL-8, (B) CaSR, (C) COX-2, and (D) PGES-1 in Caco-2^GFP­^ cells after 4h treatment with either vehicle control (0.1 % DMSO), 1 µM NPS *R*-568, CaSR ligands (5 mM Ca^2+^, and 5 mM spermine), or 5 mM spermine in combination with 1 µM NPS 2143 (CL). Mean ± SD, N = 3-5, one-way ANOVA with Tukey’s post-hoc test (0.1% DMSO).

Figure S5. PGE_2_ secretion by CaSR activation in Caco-2^CaSR-GFP^ cells. PGE_2_ secretion in supernatant of (A) Caco-2^CaSR-GFP^ and (B) Caco-2^GFP^ after 4 h treatment with either vehicle control (0.1 % DMSO) or 1 µM NPS *R*-568. Undetectable PGE_2_ concentrations were considered to be 0 pg/mL. Mean ± SD, N = 3, Mann-Whitney U test.

Figure S6. Unaltered PGE_2_ pathway genes in CaSR-ligand treated mice with colitis. Relative gene expression of (A) COX-1, (B) COX-2, (C) cPGES, (D) mPGES-1, (E) EP1, (F) EP2, and (G) EP4 in the colons of mice with DSS-induced colitis treated with CaSR allosteric modulators (each 10 mg/kg). Mean ± SD, N = 7-10. Proximal and distal colon were analyzed separately by one-way ANOVA with Dunnett’s post-hoc test *vs*. vehicle control (20% cyclodextrin), ** *p* < 0.01.

Figure S7. PGE_2_ pathway genes in CaSR-ligand treated healthy mice. Relative gene expression of (A) COX-2, (B) cPGES, (C) mPGES-2, (D) EP1, and (E) EP3 in the non-inflamed colons of a xenograft mouse model treated with CaSR modulators (each 30 mg/kg). Mean ± SD, N = 4-5. Proximal and distal colon were analyzed separately by one-way ANOVA with Dunnett’s post-hoc test *vs*. vehicle control (20% cyclodextrin).
